# Titanium nanoparticles activate a transcriptional response in Arabidopsis that enhances tolerance to low phosphate, osmotic stress and pathogen infection

**DOI:** 10.3389/fpls.2022.994523

**Published:** 2022-11-01

**Authors:** Francisco Gabriel Pérez-Zavala, Karina Atriztán-Hernández, Paulina Martínez-Irastorza, Araceli Oropeza-Aburto, Damar López-Arredondo, Luis Herrera-Estrella

**Affiliations:** ^1^ Unidad de Genómica Avanzada/Langebio, Centro de Investigación y de Estudios Avanzados, Irapuato, Mexico; ^2^ Intitute of Genomics for Crop Abiotic Stress Tolerance, Department of Plant and Soil Science, Texas Tech University, Lubbock, TX, United States

**Keywords:** beneficial elements, titanium oxide, abiotic stress, phytohormones, transcriptomic, ionomic, nutrient starvation, silicon

## Abstract

Titanium is a ubiquitous element with a wide variety of beneficial effects in plants, including enhanced nutrient uptake and resistance to pathogens and abiotic stresses. While there is numerous evidence supporting the beneficial effects that Ti fertilization give to plants, there is little information on which genetic signaling pathways the Ti application activate in plant tissues. In this study, we utilize RNA-seq and ionomics technologies to unravel the molecular signals that Arabidopsis plants unleash when treated with Ti. RNA-seq analysis showed that Ti activates abscisic acid and salicylic acid signaling pathways and the expression of NUCLEOTIDE BINDING SITE-LEUCINE RICH REPEAT receptors likely by acting as a chemical priming molecule. This activation results in enhanced resistance to drought, high salinity, and infection with *Botrytis cinerea* in Arabidopsis. Ti also grants an enhanced nutritional state, even at suboptimal phosphate concentrations by upregulating the expression of multiple nutrient and membrane transporters and by modifying or increasing the production root exudates. Our results suggest that Ti might act similarly to the beneficial element Silicon in other plant species.

## 1 Introduction

Titanium (Ti) is the second most abundant transition metal on the earth´s crust (Yaroshevsky, 2006), most of it present as titanium dioxide (TiO_2_) alone or in complex with Fe-oxides (Zhang et al., 2011). Beneficial effects of Ti application on plant growth were recognized over a hundred years ago (Traetta-Mosca, 1913; Němec and Káš, 1923). Applied to the roots or as foliar fertilizer, different chemical formulations of Ti (organic acids chelates, titanium sulfates, or directly as TiO_2_) improves crop performance by enhancing nutrient uptake, photosynthesis, growth rate, and increasing tolerance to biotic and abiotic stresses (see Lyu et al., 2017 for a review). Once Ti has entered the plant, it accumulates mainly in roots and stems independently of the chemical form applied or tissue treated (Kelemen et al., 1993), suggesting that plants are able of taking up, translocating, and sensing Ti. Although some reports suggest that Ti nanoparticles enter the plant by active transport (Chahardoli et al., 2022), the detailed mechanisms of Ti uptake, translocation, and sensing remain largely unknown. It has been speculated that Ti uptake occurs *via* iron (Fe)-transporters, thus competing with Fe to enter the plant (Cigler et al., 2010; Lyu et al., 2017). However, Ti treatment induces the transcriptional activity of some metal ion transporters-coding genes but not the classic Fe transporter IRON-REGULATED TRANSPORTER 1 (IRT1) (Tumburu et al., 2017).

Two of the most common effects of Ti application on several plant species are enhanced photosynthesis and increased nutrient uptake. These effects could be the primary origin of all other benefits of Ti such as augmented crop productivity, fruit quality, and improve tolerance to biotic and abiotic stresses, such as resistance to fungal pathogens (Jajor et al., 2021; Satti et al., 2021) and to osmotic stresses (Yin et al., 2020; Silva et al., 2022). It has been proposed that enhanced photosynthesis could be due to Ti promoting electron transport rate in the chloroplast by increasing the Hill reaction (Hong et al., 2005). Transcriptomic microarray analysis in Arabidopsis treated with TiO_2_ showed increased expression of Fe-S assembly genes (Tumburu et al., 2017) which could increase the level of the electron transport chains needed for photosynthesis (Balk and Pilon, 2011). Improved nitrogen (N), Fe, and phosphorus (P) uptake in response to Ti treatment has been reported for several plant species including wheat (*Triticum aestivum*), tomato (*Solanum lycopersicum*), soybean (*Glycine max*) and rice (*Oryza sativa*), (Zahra et al., 2017; Dağhan et al., 2020; Hussain et al., 2021; Carbajal-Vázquez et al., 2022). In the case of P uptake, the effect of Ti has been attributed to effects on root architecture (Wei et al., 2020; Hussain et al., 2021) and changes in root exudates composition, which increase soil exploration capacity and P availability (Zahra et al., 2015; Zahra et al., 2017; Zahra et al., 2019). There is little information regarding the effect of Ti treatment on the uptake of nutrients other than N, P, and Fe.

It was proposed that the effects of Ti on plants are due to hormesis, a paradoxical effect that takes place when a low dose of a toxic factor is perceived and causes a positive effect on the organism (Calabrese, 2009). Hormesis may explain why elements that are generally considered toxic like aluminum (Al), have beneficial effects on plants at low doses (Bojórquez-Quintal et al., 2017). If Ti is sensed by plants like a toxic metal, one of the expected responses is an increase in secretion of organic acids and phytochelatins, similar to what takes place in response to other toxic metals (Cobbett, 2000; Hocking, 2001; Barceló and Poschenrieder, 2002; Javed et al., 2013). Ti treatment increases the expression of Lead (Pb), Cadmium (Cd), Arsenic (As), and Aluminum-responsive genes in Arabidopsis roots (Tumburu et al., 2017) and of organic acids exudation in maize roots (Zea mays) (Ghoto et al., 2020). These compounds are known to increase nutrient availability in the soil (e.g., P and Fe) and facilitate their uptake by the plant (Gerke, 1993; Hocking, 2001; Jones et al., 2003). More recently, Ti has been found to improve plant tolerance to multiple fungal pathogens (Jajor et al., 2021; Satti et al., 2021) and to protect plants from high salinity and drought stresses (Silva et al., 2022). Despite the increasing use of Ti in agriculture (Faraz et al., 2020), the molecular mechanisms behind the beneficial effects of Ti on plant growth and productivity are still poorly understood.

In this study, we used transcriptomics and ionomics platforms to study the Ti effects and better understand the molecular basis of the beneficial effects on plant growth and tolerance to biotic and abiotic stress. We found that Ti improves shoot growth and enhances the content of essential elements including P, Copper (Cu), sulfur (S), Zinc (Zn), Fe, potassium (K), manganese (Mn), magnesium (Mg), and calcium (Ca). Our data also show that Ti-treatment increases the expression of genes involved in multiple signaling pathways involved in the tolerance to osmotic stress and pathogen infection. Moreover, we document that Ti-treated Arabidopsis plants have enhanced tolerance to high salinity, drought stress, and Botrytis cinerea infection.

## 2 Material and methods

### 2.1 Biological material

*Arabidopsis thaliana* (Col-0 ecotype; CS70000) was used as the wild-type genotype in this study. *Botrytis cinerea* was kindly provided by Dr. Alfredo Herrera-Estrella at UGA-Langebio Cinvestav (Irapuato, Mexico).

### 2.2 Growth conditions to assess the effect of Ti treatment

Arabidopsis seeds were surface sterilized by soaking seeds in 100% ethanol for seven minutes, then in 20% commercial bleach solution for seven minutes; seeds were rinsed three times with sterile distilled water and then kept in water in the dark at 4°C for two days for stratification. Arabidopsis seeds were sown onto 1% agar-0.1X Murashige and Skoog (MS) medium plates as described previously (López-Bucio et al., 2002). Seedlings were grown in a Percival chamber at 22 °C and a 16h light/8h dark photoperiod with a PAR intensity of ≈135 µmol·m^-2^·s^-1^. All experiments were carried out under these conditions unless otherwise specified.

To study the effect of Ti on Arabidopsis growth TiO_2_ nanopowder (21 nm principal particle size, 99.5% purity, Sigma-Aldrich Cat. No. 718467) was added directly to the MS medium and then the pH was adjusted to 5.7. Ti concentrations tested were 0.1, 0.250, 0.50, 1, 2, 4 and 8 mM. Different Pi concentrations (0.005, 0.05, 0.125, 0.250, 0.5, and 1 mM) were set by adding potassium monophosphate (KH_2_PO_4_) to the MS medium as required; 1 mM KH_2_PO_4_ was used as control condition. The length of the primary root were determined using ImageJ v1.51j8 (Schneider et al., 2012).

### 2.3 Free phosphate quantification

Free inorganic phosphate (Pi, ${\text{PO}}_{4}^{3-}$ ) was measured in roots and shoots of ten-day-old Arabidopsis plantlets as described by Kanno et al. (2016) with slight modifications. Briefly, 20 Arabidopsis seedlings per treatment were pooled and weighted; the material was then flash-frozen with liquid nitrogen and homogenized in a 1.5 ml Eppendorf tube with a homogenizer (CAFRAMO, model: RZR1), and then resuspended in 1ml of MES buffer (3.5 mM, pH 5.7, Sigma-Aldrich Cat. No. M8250). After centrifugation at 10,000 RFC for 10 minutes, 4 µL of the supernatant was diluted in 76 µL of MQ-H_2_O and mixed with 60 µL of 127 mM molybdate solution dissolved in 3M H_2_SO_4_ (Productos Quimicos Monterrey, Cat. No. 0540) in a 96-well plate. After ten minutes, the samples were mixed with 60 µL of 376 µM green malachite solution (Sigma-Aldrich, Cat. No. M6880). Absorbance at 610 nm was measured after 2 h of incubation using a plate reader Tecan Infinite M1000 (TECAN, Männedorf, Switzerland). The phosphate concentration was calculated per mg of tissue using a calibration curve made from 50 to 250 µM KH_2_PO_4_. All experiments were carried out with five biological replicates and three technical replicates each.

### 2.4 Collection of root exudates

To collect the root exudates of Arabidopsis seedlings, three sterile and stratified seeds were sown in 0.1X MS medium agar columns with control medium or Ti medium. Agar columns (15 mm diameter x 100 mm height) were prepared using 15 ml tubes as mold. Two agar columns (six plantlets in total) were inserted in a sterile flat-base 50 ml falcon tube and filled in with 14 ml of liquid MS medium; the tubes were covered with sterile cotton stoppers and kept in a Percival chamber at 22 °C and 16h light/8h dark photoperiod with ≈110 µmol·m^-2^·s^-1^ light intensity. Sterile Milli-Q water (Milli-Q Synthesis, Merck Millipore) was added when needed to maintain a constant liquid level. After ten days, the growth medium in each 50 ml tube was collected and concentrated in a speed vac (miVac DNA-12060-C00, Genevac) at room temperature to a final volume of 2 ml.

### 2.5 Evaluation of phytosiderophore activity using the Chrome Azurol S assay

To determine phytosiderophore activity potentially present in the root exudates, we used the Chrome Azurol S (CAS) colorimetric assay based on the modified CAS dye as developed by Alexander and Zuberer (1991). The pH of the dye was adjusted to 5.7 as in the MS growth medium. To measure the chelate activity of the root exudates, 150 µL of the concentrated root exudate was resuspended in 150 µl of the CAS dye and mixed in a 96-well plate. The reaction was incubated for two hours at room temperature and then absorbance was measured at 630 nm with a plate reader Tecan Infinite M1000 (TECAN, Männedorf, Switzerland). To have a reference for the chelate power of root exudates, a calibration curve was made with a solution of citric acid dissolved in MS medium. MS medium was used as the blank to adjust the background. Three technical replicates were averaged for each of the five biological replicates.

### 2.6 Phosphate solubilization by root exudates assays

A volume of 150 µl of root exudate was diluted to a total volume of 1 ml with MES 3.5 mM buffer pH 5.7. Then 1 mg of the insoluble phosphate salt calcium phosphate tribasic (Ca_5_HO_13_P_3_) (Cat. No. C-5267, Sigma) was added to the root exudate solution and shaken in an orbital shaker at 120 rpm for two hours at room temperature. Samples were then centrifuged for ten minutes at 20,000 rcf and 2 µl of the samples were used to determine available Pi as described above. Root exudates and Ca_5_HO_13_P_3_ in MES buffer pH 5.7 were used as controls.

### 2.7 Elemental profiling by ICP-MS

300 seedlings were germinated in a hydroponic system using liquid MS medium as reported previously by Alatorre-Cobos et al. (2014) with slight modifications. In brief, plants were sown in a 0.75 mm by 0.75 mm opening size mesh touching liquid MS medium. Five days after germination, seedlings were transferred to MS medium agar plates supplemented with 2 mM Ti or only MS medium as the control reference and grown for three or ten more days in the conditions described above. Afterward, shoots and roots were collected separately and dried at 65 °C for 5 days. Roots and shoots from a total of 1,500 plantlets per treatment were collected and pooled for each replicate. The elemental profile analysis was conducted for 19 elements in the standardized pipeline of the Baxter laboratory at the Donald Danforth Plant Science Center as described by Ziegler et al. (2013) The content of each sample was obtained as mg of the analyte/kg of the sample and used to calculate relative changes of each element; data is reported as a percentage change.

### 2.8 Preparation of RNA-seq libraries

Arabidopsis seedlings were first germinated in agar MS medium as described in the “Growth conditions to assess the effect of Ti treatment” section; five days after germination, 160 seedlings were transferred to the Ti or control treatment maintaining the same photoperiod and temperature and grown for three or ten additional days. In the case of Ti treatments, 2 mM of TiO_2_ was added to the medium, therefore samples were collected at two-time points, 3 and 10 days after Ti treatment, and from two tissues, root and shoot. We performed three biological replicates of each treatment for a total of 24 RNA-seq libraries that were named according to treatment (control: Ctrl; Ti-treated: Ti), tissue (shoot: S; root: R), and time-point (3 days: 3D; 10 days:10D). Tissue from 160 seedlings per treatment were collected separately and pooled for each biological replicate, then flash-frozen in liquid nitrogen and homogenized to isolate total RNA using TRIzol (Invitrogen, Carlsbad, CA). mRNA-seq libraries were generated using the TruSeq Illumina protocol for the pool of 160 seedlings from each of the different treatments. Each library was sequenced using a NovaSeq 6000 platform with paired-end 150bp reads. Each treatment included three biological replicates accounting for 24 libraries in total.

### 2.9 RNA-seq data analysis

Quality of sequencing reads was performed using FastQC v0.11.9 (Andrews, 2010), then reads were processed using TrimGalore v0.6.6 (Krueger, 2012) and cutaddapt (Marcel, 2011) to remove adapters and low-quality sequences. Reads abundance was quantified using the pseudo alignment program kallisto v0.44.0 (Bray et al., 2016), using the *Arabidopsis thaliana* genome Araport11 [updated 2021-06-22 (Cheng et al., 2017)] to build the index for the pseudo alignment. To integrate the transcript-level abundance estimates from kallisto into the edgeR pipeline we used the R package tximport v1.20.0 (Soneson et al., 2016). Differential expression analysis was carried out with R using the edgeR package v3.34.0 (Robinson et al., 2009); samples from different tissues were analyzed independently. The dispersions were estimated with the “estimateDisp” function and visualized with the “plotBCV” function. The common Biological Coefficient of Variation (BCV) for root was 0.1267 and for shoots 0.2163; both BCV are around the typical values for similar experiments according to the edgeR manual. Pairwise comparisons were computed with the glmLRT approach (McCarthy et al., 2012), and using a -0.2 > logFC< 0.2 cutoff value and a false discovery rate of 5%. Gene Ontology (GO) (Ashburner et al., 2011) enrichment analysis was carried out using the R package topGO v2.44.0 (Alexa and Rahnenfuhrerl, 2021). Relations between sets of DEGs were analyzed using the R package dplyr v1.0.7 (Wickham et al., 2021) and drawn with the R packages ggvenn v0.1.9 (Yan, 2021) and ggplot2 v3.3.5 (Wickham, 2009).

### 2.10 Drought stress experiments

To determine if Ti treatment ameliorates drought stress in Arabidopsis plants, we compared Ti-treated plants against control plants using the method designed by Earl (2003) with some modifications. The commercial growing substrate Sunshine mix #3 from SunGro Horticulture (http://www.sungro.com/professional-product/sunshine-mix-3/) was dried at 65 °C for three days before the experiment. Ti treatments in soil were set up by manually mixing TiO_2_ directly with the growing mix at a concentration of 159.74 ppm, which is equivalent to 2 mM TiO_2_ in agar plates. Control treatments were set up by only using growing mix, 12 pots per treatment were filled with 50 g of the growing mix as the control treatment and 12 more with the Ti supplemented growing mix. Arabidopsis seedlings were germinated onto agar plates with standard MS growth medium or MS growth medium + Ti 2 mM. Five 14-days-old seedlings were transferred to pots of their respective treatment (Control or Ti) without disturbing the soil and incubated as described above; PAR intensity was adjusted to ≈135 µmol·m^-2^·s^-1^. Seedlings were fertilized with liquid MS (0.1X) medium on the day of transplanting. On day 4 after transplanting, the pots were irrigated once with double distilled water at field capacity. After testing different periods to assess Arabidopsis’s response to drought stress, we decided to suspend watering for a period of 35 days. Afterward, all pots were re-watered to field capacity, and the plant’s survival rate was evaluated a week after. A total of 60 plants were evaluated for each treatment.

### 2.11 Salinity tolerance experiments

To assess if Ti treatment helps Arabidopsis uphold against high NaCl concentrations, we followed the method described by Corrales et al. (2014). Briefly, 60 surface-sterilized and stratified Arabidopsis seeds were sown and grown for 10 days onto 0.1 x MS medium agar plates. Two conditions were established for seed germination: MS control media (Ctrl), consisting of MS medium only, and MS + Ti, MS medium supplemented with Ti 2 mM. After 10 days of germination, seedlings were then transferred to MS medium agar plates with the same treatment but supplemented with 80 mM NaCl. Seedling’s survival rate was recorded after seven days of exposure to NaCl. To determine if a Ti exposure during germination (10 days) is enough to grant Arabidopsis NaCl tolerance, one additional treatment was set in which 60 seedlings germinated in a Ti-containing medium were transferred to a medium supplemented with 80 mM NaCl without Ti.

### 2.12 Real time quantitative PCR

Total RNA was isolated from root tissue as described in previous sections. 500 ng of RNA was used to carry out the reverse transcription reaction with SuperScript III reverse transcriptase (Invitrogen), following the manufacturer’s protocol. The RT-qPCR was performed with a MIC thermocycler (Magnetic Induction Cycler from Biomolecular Systems) using specific primers for each gene according to the qPrimer Data Base (Lu et al., 2018, https://biodb.swu.edu.cn/qprimerdb/. **Supplementary table S9**) and using the reagent SensiFast TM SYBR No-ROX Kit from Bioline. The PCR conditions were 95°C for 2 min, followed by 40 cycles of 95°C for 5 sec, 65°C for 10 sec and 72°C for 20 sec. The relative expression of each gene was calculated relative to the geometric mean of the Cq of the ACTIN2 (ACT2) and UBIQUITIN-CONJUGATING ENZYME 21 (UBC21) genes for each sample.

### 2.13 Assessment of the effect of Ti against fungal interactions

To assess the effect of Ti on Arabidopsis responses when challenged with *Botrytis cinerea*, experiments in substrate with adult plants were performed. Surface-sterilized and stratified seeds were germinated under two different conditions: MS control media (Ctrl), consisting of MS medium only, and MS + Ti (2 mM), MS medium supplemented with Ti. After 10 days, plantlets were transplanted to commercial growing mix Sunshine mix #3 from SunGro Horticulture and incubated in a Percival chamber as mentioned previously. Ti treatments in substrate were made as explained above for drought experiments. 10 days after transplanting, leaves of Arabidopsis plants were inoculated with 10 µL of *B. cinerea* conidia (1×10^4^/µL). Photographs were taken at one, three, and seven days after the inoculation, and the number of leaves with necrotic spots and area of the lesion were analyzed with ImageJ v1.51j8 (Schneider et al., 2012). To investigate if exposure to Ti during germination is enough to confer enhanced resistance to *B. cinerea* without additional Ti exposure, one additional treatment was set up in which seedlings were germinated in a Ti-containing medium, and after 10 days they were transferred to soil without Ti supplementation. A total of 25 leaves per time and treatment were analyzed.

## 3 Results

### 3.1 Beneficial effects of Ti in Arabidopsis growth are dose-dependent under Pi sufficiency

**Figure 1:**
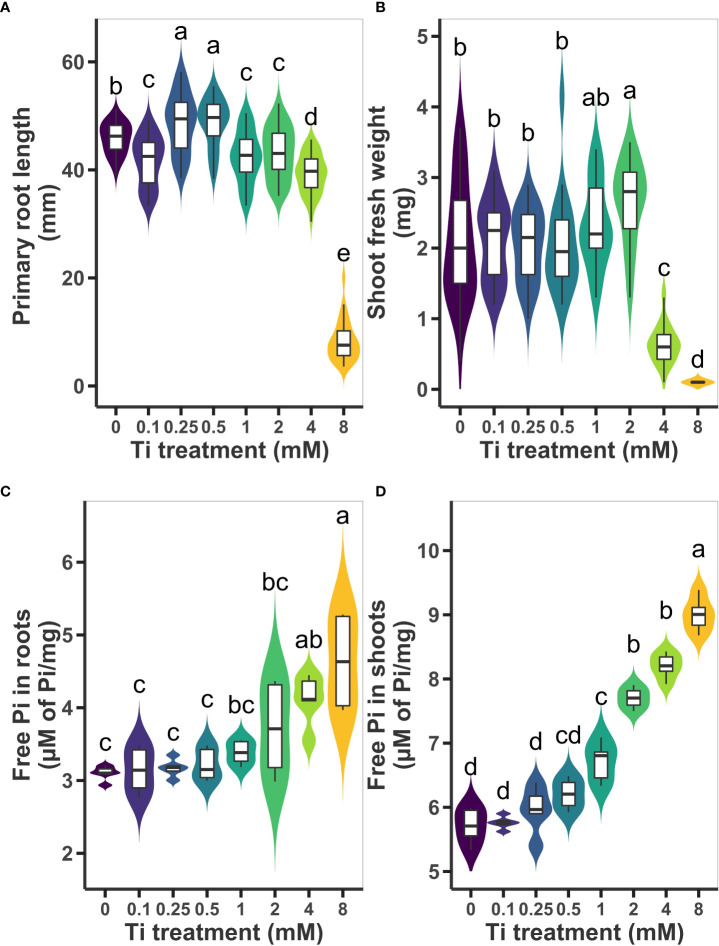
Effect of Titanium (Ti) on Arabidopsis growth and inorganic phosphate (Pi) content. **(A)** Primary root length and **(B)** shoot fresh weight were determined ten days after treatment with 0.2, 0.25, 0.5, 1, 2, 4, and 8 mM Ti. Concentration of free Pi was also determined in roots **(C)** and shoots **(D)** from experiments in **(A, B)** No added Ti (0) was used as control treatment. Different letters indicate statistically significant differences according to Tukey’s significance test (p< 0.05).

To study the effects of Ti treatment on Arabidopsis seedlings, we first determined an effective Ti concentration that results in beneficial effects to seedlings grown in MS medium. We evaluated shoot fresh weight, primary root length. We decided to also use free inorganic phosphate (Pi) as a marker of enhanced nutrient uptake, therefore, we measure Pi content in shoot and root 10 days after treatment with different concentrations of Ti (0, 0.1, 0.25, 0.5, 1, 2, 4, and 8 mM). We observed that moderate Ti concentrations (0.25 and 0.5 mM) caused an increase of the primary root length by 6.2%, whereas higher Ti concentrations (1, 2, 4 mM) caused a slight inhibition of 7.4%, 5.8%, 14.4%, respectively, in reference to untreated plants (**Figure 1A**). The highest Ti concentration, 8 mM, severely compromised root growth. Unexpectedly, we found that 0.1 mM Ti also had an inhibitory effect on primary root length (**Figure 1A**). In the case of shoot fresh weight, low concentrations of Ti had no significant effect; 1 mM had a reproducible but no significant effect in increasing shoot biomass whereas 2 mM caused an increase of 26.7% in shoot biomass. High concentrations of Ti (4 and 8mM) had a drastic inhibitory effect on shoot biomass accumulation (**Figure 1B**). Pi in the root increased only at Ti concentrations of 4 and 8 mM (**Figure 1C**). Accumulation of free Pi in Arabidopsis shoot did not change significantly at Ti concentrations below 0.5 mM. However, free Pi increased in shoots by 17%, 34%, and 43% at concentrations of 1, 2, and 4, mM, respectively (**Figure 1D**). Due to its positive effect on shoot Pi content and shoot biomass accumulation without causing primary root growth inhibition (**Figure 1B**), we chose a 2 mM Ti concentration for further experiments.

### 3.2 Ti treatment improves Pi uptake in Pi-starved Arabidopsis seedlings

**Figure 2:**
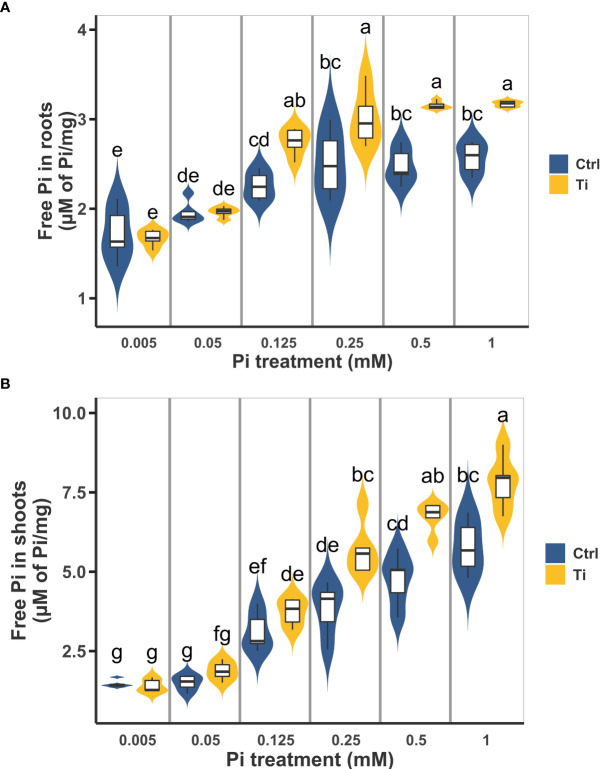
Effect of Titanium (Ti) on Arabidopsis inorganic phosphate (Pi) content under variable Pi conditions. Arabidopsis seedlings were grown under 0.005, 0.05, 0.125, 0.5, and 1 mM Pi and treated with Ti 2 mM. Free Pi content was determined in root **(A)** and shoot **(B)** after ten days of germination. No added Ti was used as control treatment. Different letters indicate statistically significant differences according to Tukey’s significance test (p< 0.05).

To explore in more detail the effect of Ti treatment on Pi accumulation in Arabidopsis seedlings, we evaluated the shoot and root Pi content of seedlings subjected to six different Pi concentrations ranging from 0.005 mM to 1 mM with or without Ti treatment (2 mM). As expected, in control plants Pi content in root and shoot increased with the increased concentration of this nutrient in the media, reaching a maximum of free Pi at 0.5 mM Pi for the shoot and 0.25 mM for the root. Pi content was statistically higher in the root of Ti-treated plants at 0.125 mM and higher Pi concentrations than in untreated plants (**Figure 2A**). A similar effect was observed in the case of the shoot with Pi content as significantly higher in Ti-treated plants at 0.25 mM and higher Pi concentrations (**Figure 2B**). Importantly, Pi content in roots of seedlings grown under 0.125 mM Pi and treated with Ti is comparable to that of roots of control plants grown at 1 mM Pi. Pi content in shoots of plants grown at 0.25 mM Pi in media containing Ti was similar to that of plants grown in 1mM without Ti treatment (**Figure 2B**). These results agree with previous reports that Ti treatments enhance nutrient uptake under optimal and sub-optimal nutrient levels.

To gain insights into the potential mechanisms triggered by Ti treatment to improve P status in Arabidopsis, we tested if the root exudates from Ti treated and control plants have different capacities to solubilize Pi and/or chelate metallic cations that compromise Pi availability. Root exudates of treated (2 mM Ti) and untreated plants were recovered to evaluate their capacity to solubilize calcium tribasic phosphate (Ca_5_HO_13_P_3_), a P salt practically insoluble in water. We also tested for phytosiderophore activity using a Chrome Azurol S (CAS) colorimetric assay (see Materials and Methods). We observed that the Pi solubilizing capacity of the root exudates of Ti-treated plants was 20% higher than that of control plants and had almost 3 times more Fe-chelating activity than those produced by untreated control plants (**Supplementary Figure 1**).

### 3.3 Ti treatment improves the overall nutritional state of treated plants

**Figure 3:**
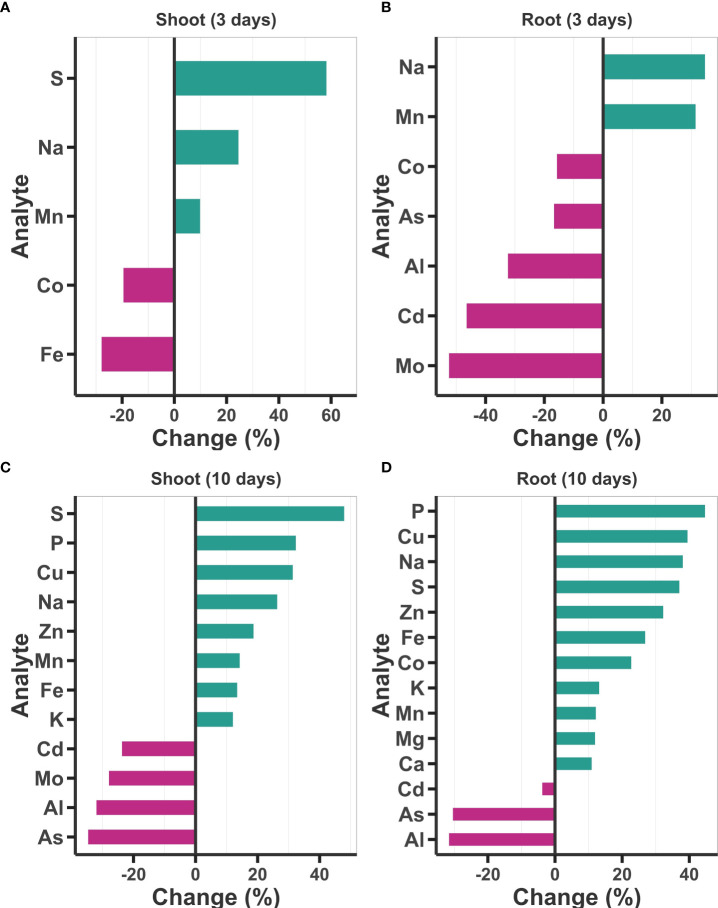
Effect of Titanium (Ti) on elemental content of Arabidopsis. Content (mg of the analyte/kg of the sample) of 19 elements was determined in shoot and root three days **(A, B)** and 10 days **(C, D)** after the treatment with Ti 2 mM. Only elements with statistically significant changes according to Tukey’s significance test (*p<* 0.05) are presented. Changes are presented as percentage changes.

To determine whether treatment with Ti has an impact on the accumulation of elements other than N and P in the plant, we performed an ICP-MS ionomic profiling of 19 elements (B, Na, Mg, Al, P, S, K, Ca, Mn, Fe, Co, Ni, Cu, Zn, As, Rb, Sr, Mo, and Cd). This analysis revealed that Ti induces important changes in the elemental composition of Arabidopsis seedlings even three days after treatment (**Figure 3**). In the shoot of plants treated for 3 days with Ti, three elements S (58%), Na (23%) and Mn (10%) had increased content compared with the controls, while a decrease in Fe (19.47%) and Co (27.82%) was also detected in Ti treated plants (**Figure 3A**). In the case of roots treated with Ti for three days, they had 31.46% Na and 34.46% Mn higher content than the control plants, whereas five elements (Co, As, Al, Cd, and Mo) showed a decreased content ranging from 15.72% to 52.44%, in reference to the control plants (**Figure 3B**). The elemental profile completely changed on day 10 of Ti treatment in both tissues. In the case of shoot, the content of eight essential elements (S, P, Cu, Na, Zn, Mn, Fe, and K) increased, whereas the content of four elements (Cd, Mo, Al, and As) was reduced (**Figure 3B**). Content of P, Cu, Na, S, Zn, Fe, K, and Mn increased in roots and shoots, all essential nutrients for plant growth and development, whereas the content of Cd, As and Al, which are toxic elements for plant development, were reduced significantly as compared to control plants (**Figures 3C, D**). Our data suggest that Ti treatment, in general, enhances the content of essential elements for plant growth and development, while decreasing the uptake of toxic elements.

### 3.4 Differential expression profiling suggests that Ti induces extensive transcriptomic responses in Arabidopsis in the root but not in the shoot

To gain insights into the molecular responses of Arabidopsis to Ti, we performed RNA-seq analysis in shoot and root of seedlings grown under Pi sufficiency (1 mM) and treated with Ti (2 mM). Previous microarray studies found that there is differential gene expression at seven days of exposure to Ti in Arabidopsis (Landa et al., 2012). Our results showed that three days after the treatment there is already a change in elemental content of Ti-treated plants and that at 10 days after treatment plants show evident phenotypic differences and enhanced nutrient accumulation. This suggests that transcriptional changes might take place few days after Ti treatment. Therefore, samples were collected at two time points, three and 10 days after Ti treatment to capture earlier transcriptional responses to Ti exposure. We performed three biological replicates of each treatment for a total of 24 RNA-seq libraries that were named according to treatment (control: Ctrl; Ti-treated: Ti), tissue (shoot: S; root: R), and time-point (3 days: 3D; 10 days:10D) (see Materials and Methods). Quality and alignment statistics of the different RNA-seq libraries are presented in **Supplementary Table S1 and Supplementary Table S2**. The multidimensional scaling (MDS) exploratory analysis showed little dispersion among the libraries. Both shoot and root libraries are clustered by time-point (three and 10 days) as expected, but only at day 10 libraries clustered by treatment (**Supplementary Figure S2**), suggesting that Ti-treated seedlings do not activate major gene expression changes at day three, volcano plots of the results of the RNA-seq analysis are also presented in the **Supplementary Figure S3**.

We then performed analyses to determine differentially expressed genes (DEGs) between treatments from the same tissue at the same time-points tested. First, we determined the differential gene expression profiles in response to Ti (Ti vs Ctrl) by using likelihood ratio tests (McCarthy et al., 2012) and using a -0.2 > logFC< 0.2 threshold and false discovery rate of 5% (p-values< 0.005466 for roots and< 0.000346 for shoots). After three days, we found a very discrete number of DEGs in both shoots and roots samples. In the case of shoots only one gene was detected as upregulated, AT1G07730, putatively coding for a Disease resistance-responsive (Dirigent-like protein) family protein while two genes were downregulated, AT1G24800 (F-BOX family protein) and AT4G11485 (LOW-MOLECULAR-WEIGHT CYSTEINE-RICH 11, LCR11). In the case of roots, we found that four genes were upregulated: AT3G01500 (CARBONIC ANHYDRASE 1, CA1), AT3G48200 (transmembrane protein), AT5G38420 (RUBISCO SMALL SUBUNIT 2B, RBCS2B), and AT5G51585 (transmembrane protein); and only one downregulated gene, AT3G47340 (GLUTAMINE-DEPENDENT ASPARAGINE SYNTHASE 1, ASN1). The rather mild response of Arabidopsis to Ti after 3 days of exposure suggests that Ti uptake and translocation is rather slow or that the plant does not directly react to the presence of Ti but to a signaling molecule produced when Ti accumulates above a threshold in the plant.

**Figure 4:**
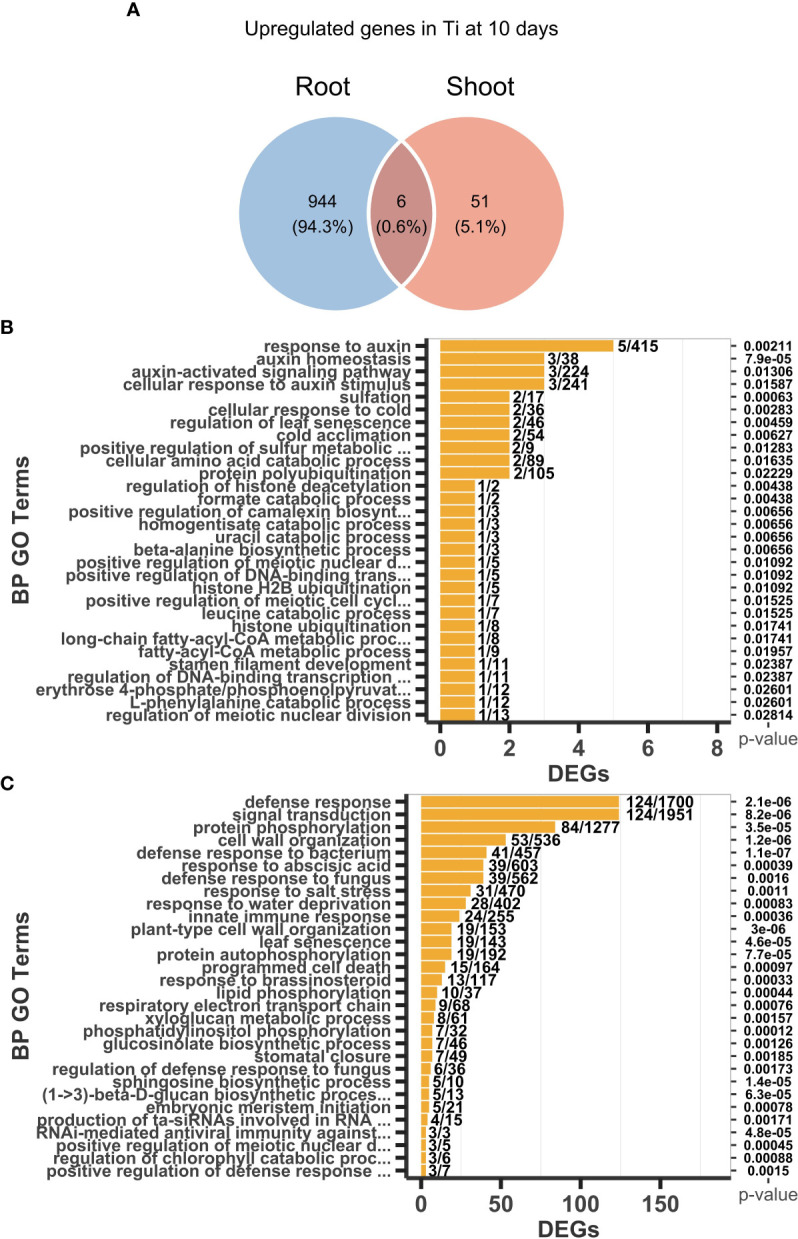
Transcriptomic responses and enriched Gene Ontology (GO) categories of differentially expressed genes (DEGs) in response to Titanium (Ti) in Arabidopsis. **(A)** Shared upregulated DEGs between roots and shoots. Top 30 enriched GO categories of upregulated DEGs in roots **(B)** and shoots **(C)** in response to Ti treatment.

After 10 days of Ti treatment, 950 and 1277 were detected as up and downregulated genes, respectively, in the root. We also determine that 57 and 79 genes were up and downregulated, respectively, in shoots. Venn analysis helped us to determine sets of genes that are common or uniquely upregulated and downregulated at 10 days across the two tested tissues. In the case of upregulated genes, only six genes are shared, whereas 944 and 51 are specific for root and shoot, respectively (**Figure 4A**). In the case of downregulated genes, 20 genes were shared, whereas 1257 and 59, were specific for root and shoot, respectively (**Supplementary Figure S4**, **Supplementary Tables S3–S6**). These data suggest that Ti treatment causes changes in the transcript level of a large number of genes in the roots, while it causes changes in transcript level in only a few genes in the shoot, and that the responses to Ti seem to be mainly organ specific.

### 3.5 GO analysis revealed the activation of biotic and abiotic stress responses in both shoot and root in response to Ti

To further characterize the biological processes that are activated in response to Ti treatment, we manually analyzed DEGs induced at three days of Ti treatment and performed a Gene Ontology (GO) enrichment analysis of DEGs induced 10 days after the treatment with Ti. In the case three days after the treatment, we found that two of the four upregulated genes in roots of Ti-treated plants (CA1 and RBCS2B) are targets of OXIDATIVE STRESS 2 (OXS2). OXS2 is a tandem zinc finger transcription factor previously identified to play a crucial role in salt tolerance in Arabidopsis (Jing et al., 2019). The third gene, AT3G48200, encodes a transmembrane protein which knockout mutant is tolerant to heat stress and insensitive to abscisic acid (ABA), a phytohormone playing an important role in osmotic stress responses (Luhua et al., 2013). The fourth Ti-responsive gene at three days of Ti treatment, AT5G51585, is still uncharacterized. These data suggest that Ti treatment might initially induce only genes involved in tolerance to osmotic stress.

GO analysis of upregulated genes in the root showed that GO categories enriched at 10 days of Ti treatment are related to defense responses to biotic stresses (GO:0042742 defense to response to bacterium, GO:0071555 cell wall organization, GO:0006952 defense response, GO:0045087 innate immune response, GO:0050832 defense response to fungus, GO:1900150 regulation of defense response to fungus) and responses to osmotic stresses (GO:0009414 response to water deprivation, GO:0009651 response to salt stress) (**Figure 4B**). Among the upregulated genes in roots, we found 23 genes coding for disease resistance proteins belonging to the nucleotide binding site-leucine rich repeat (NBS-LRR) family, which are receptors involved in the detection mechanism of multiple pathogens (DeYoung and Innes, 2006). We also found the upregulation of CALMODULIN BINDING TRANSCRIPTION ACTIVATOR 2, 3, and 5 (CAMTAs) and WRKY70 (**Supplementary Table S3**), which act as suppressors of the salicylic acid (SA) and jasmonic acid (JA) signaling routes respectively; both phytohormones closely related to pathogen defense responses (Li et al., 2004; Sun et al., 2019). Together, these data suggest that Ti sensitizes transcriptional responses involving a large number of genes related to pathogen defense, which expression may further be increased once the pathogen is perceived. Cell wall organization is another GO category (GO:0071555) that is enriched in the transcriptional response elicited by Ti in roots. Cell wall modifications could lead to increased rigidity of the cell wall and callose deposition that reduce the ability of pathogens to penetrate the cell wall, thus, reducing the severity of pathogen attacks.

Enriched GO categories in upregulated DEGs in roots also relate to phytohormones and phytohormone signaling pathways involved in defense responses GO:0009741 response to brassinosteroid and GO:0009737 response to abscisic acid (ABA). Both ABA and brassinosteroids are involved in alleviating stress symptoms and improving tolerance to diverse biotic and abiotic stresses including drought (Wang, 2012; Fernando & Schroeder, 2016; Rattan et al., 2022). However, we also found upregulated negative regulators of the ABA signal transduction pathway such as the GLOBAL TRANSCRIPTION FACTOR GROUP E 11 (GTE11) and the PROTEIN WITH THE RING DOMAIN AND TMEMB_185A 1 (PPRT1) (Misra et al., 2018; Liu et al., 2020). Both transcription factors negatively regulate responses to various stresses, suggesting that Ti activates the response of genes involved in biotic and abiotic stress, but without turning on the complete response pathways until the stress is perceived. Therefore, it seems that Ti activates stress responses but also represses part of the signaling pathways to prevent a penalty in plant fitness due to a high level of constitutive activation of stress responses.

In the case of shoots, the more significant and enriched GO categories 10 days after Ti treatment were related to auxin and camalexin, i.e., GO:0010252 auxin homeostasis, GO:0009733 response to auxin, GO:0071365 cellular response to auxin stimulus, and GO:1901183 positive regulation of camalexin biosynthesis (**Figure 4C**). It is worth mentioning that the number of genes in the shoot enriched GO terms are small but significant and mainly related to the regulation of general cellular processes, whereas the enriched GO terms in roots span responses against multiple biotic and abiotic stresses. Also in shoots, AT4G27260 (WES1) is one gene that is noteworthy as it has been demonstrated that it positively regulates the major camalexin biosynthesis genes, which are critical to the defense against pathogens (Zhang et al., 2007; Wang et al., 2012). Lists with the top 100 more significant GO terms from roots and shoots are presented in the **Supplementary table S7 and S8**

### 3.6 Ti treatment increases the expression of multiple nutrient uptake and nutrient starvation responsive genes

**Figure 5:**
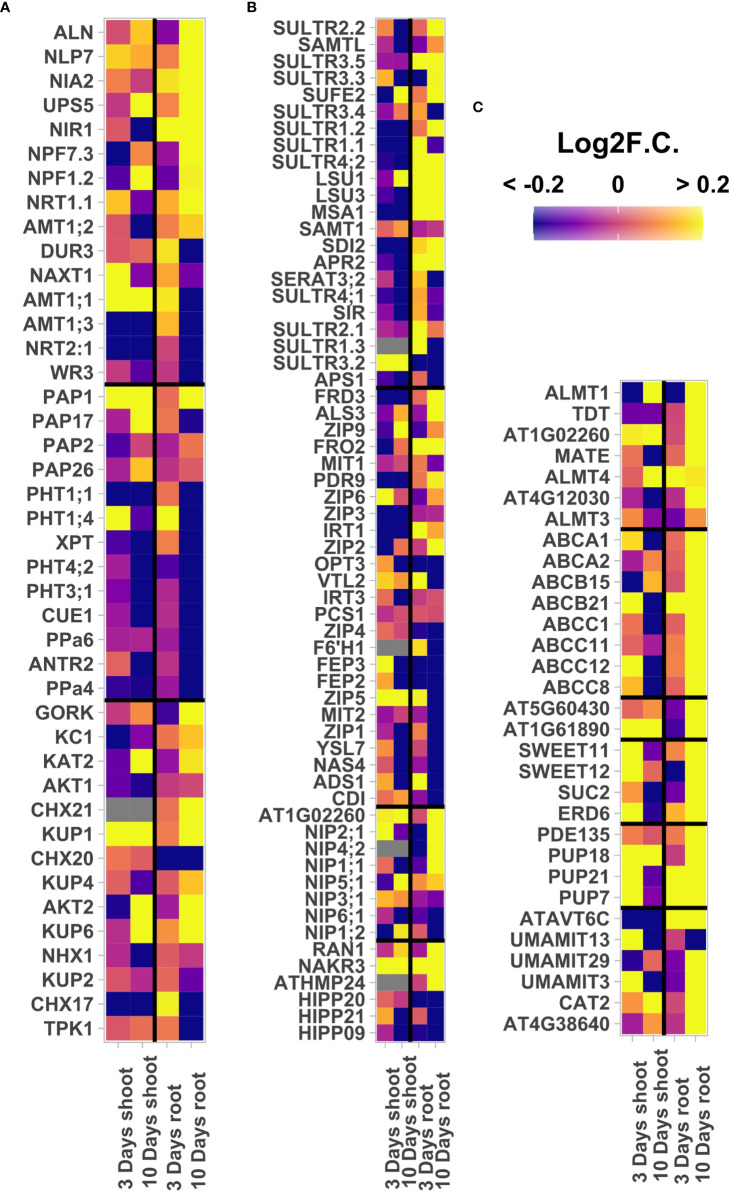
Effect of Titanium (Ti) on expression of nutrient acquisition related genes. Expression level (mean log2F.C.) of genes encoding transporters of **(A)** macro and **(B)** microelements, and other related molecules **(C)**. Gray squares indicate that the gene was filtered-out in the filtering step of the edgeR pipeline due to low CPMs. All heatmaps share the same color scale.

Increased nutrient uptake is a commonly reported beneficial effect of Ti supplementation (Kleiber and Markiewicz, 2013). While the GO analysis did not show enriched categories related to nutrient uptake, we found several upregulated DEGs related to nutrient transport or responsive to nutrient starvation in our transcriptomic data 10 days after Ti treatment. These genes include multiple upregulated genes in response to Ti encoding S, Boron (B), putative Silicon (Si) and amino acid transporters, that could play a role in enhancing nutrient uptake in response to Ti treatment (**Figure 5**). We found that 31 of the so-called Pi-starvation responsive (PSR) genes (Ojeda-Rivera et al., 2020) were upregulated DEGs in roots after 10 days of Ti treatment (**Supplementary Figure S5**). PSR genes activated by Ti treatment included those encoding the purple acid phosphatases PAP1, and PAP2, which facilitate Pi uptake from organic molecules present in soil (**Figure 5A**). Regarding N, we found that genes involved with N sensing, recycling, and transport such as ALN, NLP7, NIA2, UPS5, NIR1, NPF7.3, NPF1.2, NRT1.1, and AMT1;2 showed a change in transcriptional activation in response to Ti close to or greater than 0.2 log2F.C., which is close to an increase of 20% the expression levels (**Figure 5A**). DEGs related to K transport like GORK, KC1, KAT2, CHX21, KUP1, KUP4, AKT2, KUP6, and NHX1 (Wang and Wu, 2013) are also upregulated (log2F.C. ≥ 0.2) more than 20% (**Figure 5A**). In the case of some micronutrients, 12 Sulfur transporters are upregulated, as well as seven divalent ion transporters (Claus et al., 2013; Gigolashvili and Kopriva, 2014; Tsai and Schmidt, 2017). Heavy metals transporters (de Abreu-Neto et al., 2013; Li et al., 2020) are up and down-regulated as well as aquaporins that are B transporters and putative Si transporters and one divalent ion symporter (AT1G02260) also a putative Si transporter (Ma and Yamaji, 2015; Vatansever et al., 2017) (**Figure 5B**). ALMT1, TDT, AT1G02260, and MATE, which are organic acids transporters genes which play a role in Al detoxification thereby enhancing Pi availability, are upregulated. ALMT4, AT4G12030, and ALMT3 are also upregulated. Nine ABC transporters, two drug transporters, four sugar transporters, four nucleobase, and five amino acid transporters are also upregulated (**Figure 5C**). These data correlate with the improved content of P, S, K and divalent cations we observed and are in line with previous reports that Ti increases the uptake of P and K (Mustafa et al., 2021).

### 3.7 Ti enhances tolerance to osmotic stresses in Arabidopsis through activation of ABA- related and ROS copping genes

**Figure 6:**
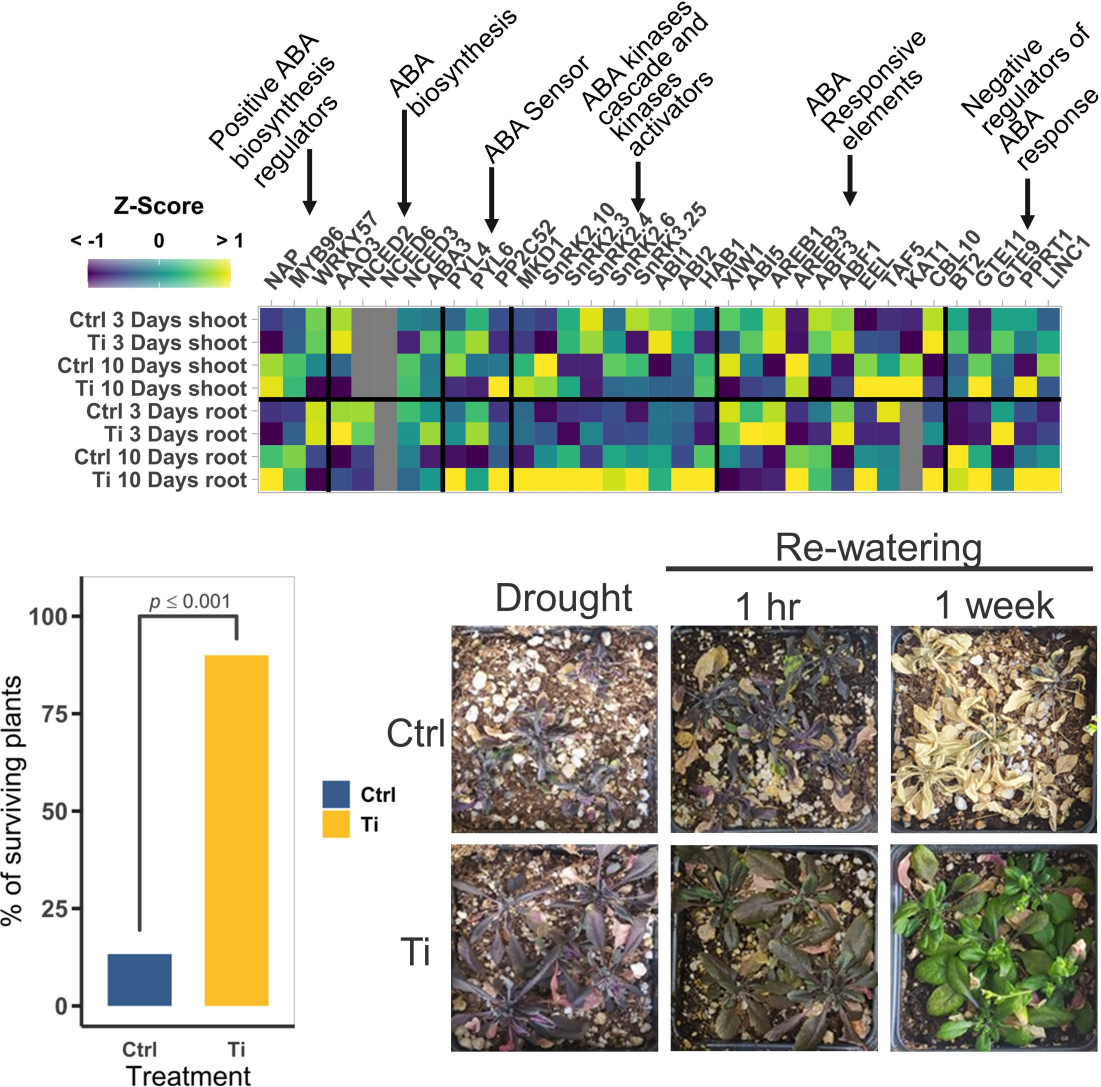
Effect of Titanium (Ti) on the expression of abscisic acid (ABA) and drought tolerance related genes in Arabidopsis. **(A)** Mean normalized expression of ABA related genes. Gray squares indicate that the gene was filtered-out in the filtering step of the edgeR pipeline due to low CPMs. **(B)** Percentage of surviving plants seven days after rewatering drought-stressed plants (n=60). **(C)** Representative photographs of plants exposed to drought before re-watering, and one hour and one week after rewatering.

In our RNA-seq analyses, we found several enriched GO categories related to ABA, water deprivation, and salt stress. Among the ABA-related genes that have a higher transcript level in Ti-treated plants than in the controls, we can highlight NAP, one of the transcription factors involved in activating ABA-biosynthesis genes, components of the ABA sensor system like PYL4, PYL6, and PP2C52 and SNRK2s kinases that is upregulated in roots but not in shoots. However, ABA-synthesis genes do not seem to be affected by Ti treatment (**Figure 6A**). Our transcriptomic data also showed that Ti-treated plants have elevated transcript levels of genes encoding ROS homeostasis proteins such as PEROXIDASE 64 (PER64), CATALASE 3 (CAT3), ALTERNATIVE OXIDASE 1A (AOX1A), and members of the Peroxidase family protein AT1G71695, AT2G37130, and AT4G30170. Additionally, we detected 30 upregulated DEGs corresponding to the GO category response to oxidative stress in roots (Top 54 GO term, *p* = 0.00635; **Supplementary Table S7**).

To test whether Ti treatment could enhance osmotic-stress tolerance in Arabidopsis, we subjected Arabidopsis plants to drought and salt stresses. In the case of drought stress experiments, 14-day-old Arabidopsis seedlings were transferred to pots with substrate previously amended with a concentration of 159.74 ppm of TiO_2_, incubated in a growth chamber, and watered regularly (see Materials and Methods). 18 days after transplanting, watering was completely stopped, and plants were subjected to drought stress for 35 days, then plants were rewatered and seven days after the recovery irrigation the number of recovered plants counted. We found that 90% of the plants treated with Ti survived the drought treatment whereas only 13% of the non-treated plants were able to recover (p ≤ 0.001) (**Figure 6B**). Interestingly, signs of recovery were visible in Ti-treated plants after one hour of re-watering (**Figure 6C**).

**Figure 7:**
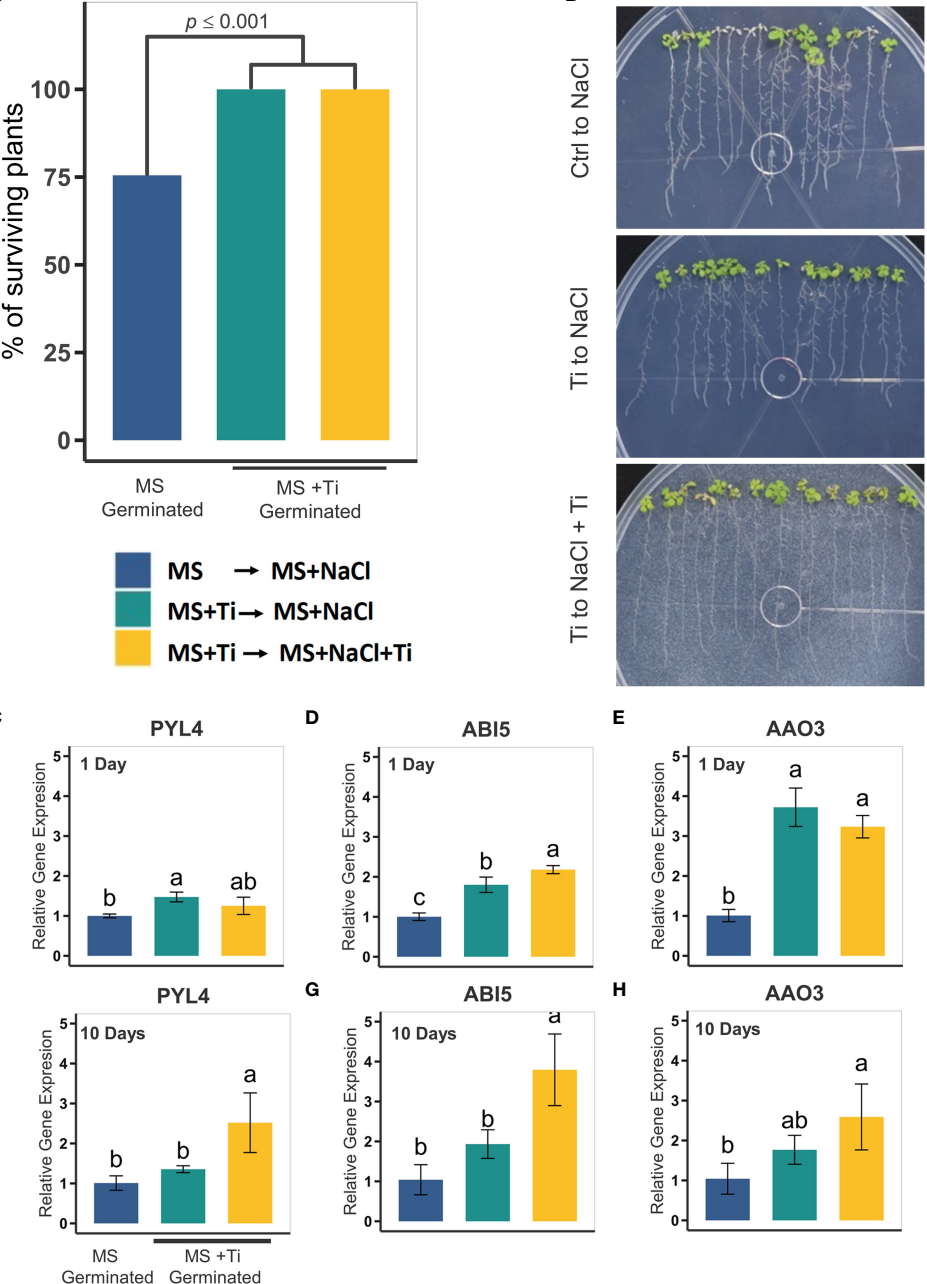
Effect of Titanium (Ti) on Arabidopsis tolerance to high salinity. **(A)** Percentage of surviving plants seven days after exposure to 80 mM NaCl. Plants were germinated in MS and then transferred to MS containing 80 mM NaCl (blue bars). Plants treated with Ti were germinated in MS medium containing 2 mM Ti and then transferred to MS media with 80 mM NaCl (green bars) or MS media containing 80 mM NaCl and 2 mM Ti (yellow bars). **(B)** Representative photographs of plants under each treatment seven days after salt-stress exposure. Relative expression of salt-stress related genes in Arabidopsis in roots: PYL4 **(C)**, ABI5 **(D)**, AAO3 **(E)** one day after Ti treatment; and PYL4 **(F)**, ABI5 **(G)**, and AAO3 **(H)**. Different letters indicate statistically significant differences according to Tukey’s significance test (*p*< 0.05).

To test whether Ti treatment also improves plant resilience to salt stress, we carried out *in vitro* experiments to evaluate Arabidopsis survival after NaCl treatment. Arabidopsis seeds were germinated in standard MS medium with or without Ti, and then transferred to MS with 80 mM NaCl only, or 80 mM NaCl in combination with 2 mM Ti (**Figure 7**). We found that when seedlings were subjected to salt stress with no Ti treatment, only 75% of the seedlings survived (p ≤ 0.001). By contrast, seedling survival increased to 100% (p ≤ 0.001) when Ti was provided at germination and during salt stress (**Figure 7A**). To test whether Ti may have a priming-like effect that increases the plant survival under salt stress, we tested plants germinated with Ti that were transferred to high NaCl media without Ti. Survival rate and phenotype were like that observed when Ti was provided before and during the stress (**Figures 7A, B**). This suggests that Ti accumulated in shoot and root serves as a protectant probably by sensitizing signaling pathways related to ABA to cope with high salinity stress.

Since pretreatment with Ti enhances tolerance to osmotic stress, we decided to determine whether the effect of Ti on the expression of genes involved in abiotic stress tolerance is maintained after transfer to media with a specific stress in the absence of Ti. With this aim, plants germinated in MS medium with 2 mM Ti for 10 days, and then transferred for 1 and 7 days to MS with 80 mM NaCl only, or 80 mM NaCl and 2 mM Ti (**Figures 7C–H**). RNA was extracted from plants subjected to the different treatments 1 and 7 days after transfer to media containing NaCl. We used RT-qPCR to determine the transcript level of PYL4, ABI5 and AAO3 1 and 7 days after transfer to media containing NaCl in the presence and absence of Ti. We found that after 1 day of transfer to media containing NaCl lacking Ti, the transcript levels of these three genes were higher in seedlings pretreated with Ti than those of the untreated controls, and similar to those present in seedlings transferred to media containing NaCl and Ti, except for PYL4. After 7 days of transfer to media containing NaCl, the transcript levels of PYL4, ABI5 and AAO3 in seedlings pretreated with Ti were higher, but not statistically different, from those present in untreated controls. Transcript levels of the three genes in seedlings transferred to media containing NaCl and Ti were significantly higher than in the untreated controls. These results suggest that the effect of Ti on the expression of genes involved in osmotic stress is maintained after 1 day of growth in the absence of this beneficial element and also that the response is greater when Ti is present when the plant is exposed to NaCl.

### 3.8 Ti treatment helps Arabidopsis to cope with *B. cinerea* infection

**Figure 8:**
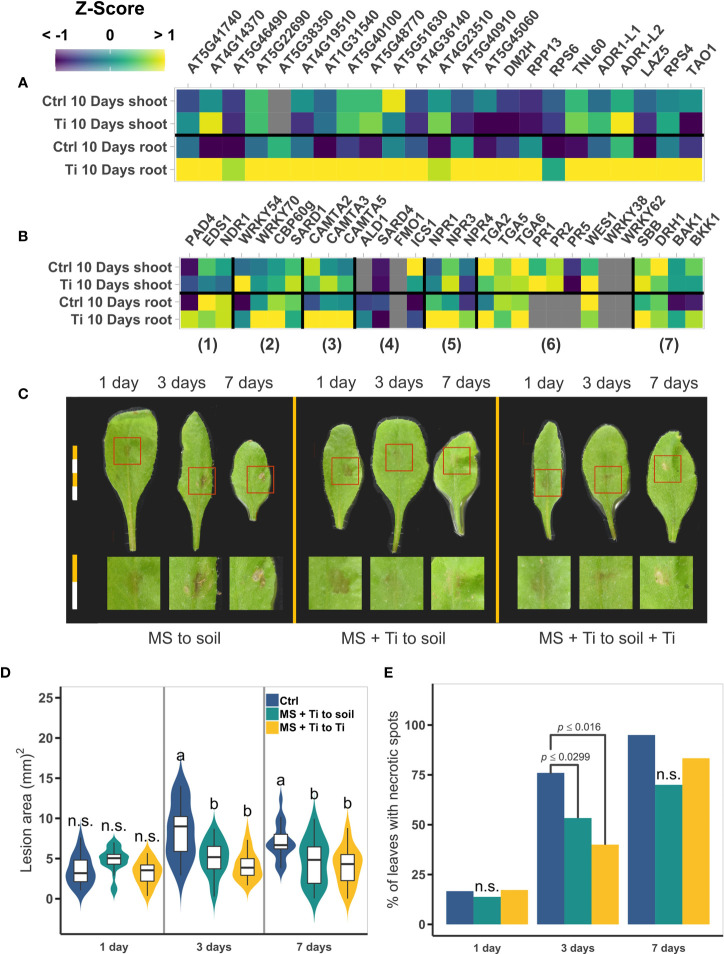
Effect of Titanium (Ti) on the expression of pathogen responsive genes and on Arabidopsis*-Botrytis cinerea* interaction. **(A)** Mean normalized expression levels of NBS-LRR receptors genes. **(B)** Mean normalized expression levels of salicylic acid (SA) and systemic acquired response related genes. Numbers bellow the heatmap indicate the function of the genes; 1) SA regulatory modules, 2) SA biosynthesis positive regulators, 3) SA biosynthesis negative regulators, 4) SA biosynthesis, 5) SA receptors, 6) SA responsive genes, and 7) cell death. **(C)** Representative photographs of plants under each treatment at one, three, and seven days after *B. cinerea* infection. Each segment of a bar are equal to 2.5 mm. Individual leaves were cropped from the original photograph to make the composition; no further modifications or picture enhancement were made. **(D)** Area of the lesion (mm^2^) in *B. cinerea* infected plants. Treatments with the same letter are not significantly different. n.s.: not significant. Different letters indicate statistically significant differences according to Tukey’s significance test (p< 0.05). **(E)** Percentage of leaves with necrotic spots. Only *p*-values of the two-tail chi-square test are presented when the values are statistically significant. In **(A)** and **(B)**; gray squares indicate that the gene was filtered-out in the filtering step of the edgeR pipeline due to low CPMs. Both heatmaps share the same color scale. Both **(D)** and **(E)**, share the same color key.

Analysis of the RNA-seq data of Ti-treated plants revealed enriched GO categories related to defense responses to bacteria, fungus, and viruses, as well as programmed cell death (**Figure 4**). Therefore, we decided to explore in more detail the effect of Ti treatment on the transcript level of genes involved in SA biosynthesis and signaling routes. Our data showed that several SA-related molecular components were responsive to Ti treatment in both shoot and roots. We found that several NBS-LRR receptors are upregulated in Ti-treated roots but only a few in shoots (**Figure 8A**). PAD4, which is part of the SA regulatory module is upregulated in roots and lightly in shoots. The positive regulators of SA biosynthesis WRKY54, WRKY70, and CBP60g, are upregulated in roots, and WRKY54 only in shoots. The negative regulators of SA biosynthesis CAMTA2, CAMTA3, and CAMTA5 are also upregulated in roots with only CAMTA3 faintly upregulated in shoots. Interestingly no SA biosynthesis genes were found upregulated either in roots or shoots, suggesting that Ti might preactivate positive regulators of SA biosynthesis but at the same time negative regulators to prevent an undesirable high level of SA that could affect plant growth. NPR1 and NPR3, which regulate the SA response pathway are upregulated in roots but only NPR3 is upregulated in shoots. Downstream of SA perception by NPR1, pathogen-related genes, and WRKYs transcription factors are activated directly by TGA2, TGA5, and TGA6, which ultimately activate the global plant immune response known as SAR. TGA2 and TGA6 are upregulated in shoots while PR1 and WES1 are upregulated in the shoot. The cell death positive regulator SBB1 is upregulated in both roots and shoots whereas the mRNA exporter DRH1 which is needed to initiate cell death, is not. We also found the receptors BAK1 and BKK1, negative regulators of SA-dependent cell death, are down-regulated in roots but not in shoots (**Figure 8B**). These results altogether suggest that Ti might induce a molecular priming-like response against pathogens.

To test whether Ti treatment improves Arabidopsis tolerance to biotic stress, we challenged Arabidopsis seedlings with the pathogen *B. cinerea*. Seed germination was performed in MS medium for the control treatment (Ctrl) and with 2mM Ti for Ti treatments (+Ti). 14-day-old Arabidopsis seedlings were then transplanted to the substrate previously amended with Ti in the cases of +Ti and without Ti for Ctrl treatments. Seven days after transplanting, leaves of Arabidopsis plants were inoculated with *B. cinerea* conidia (see Materials and Methods). To determine if Ti could have a chemical priming effect, one additional treatment was set up in which plants germinated in Ti treatment were transplanted to soil with no additional Ti. Photographs of the leaves were taken one, three, and seven days after the inoculation, and the area of the lesion and percentage of necrotic leaves was determined (see Materials and Methods). Plants treated with Ti presented lesions that were smaller and less intense relative to non-treated plants (**Figure 8C**). The lesion area is significantly smaller for the Ti-to-control and Ti-to-Ti treatments than in the control-to-control plants at both 3 and 7 days postinfection (**Figure 8D**). The progress of the infection also seems to be slower in Ti-treated plants as fewer leaves develop necrotic spots at three days, but at 7 days postinfection no statistically significant differences are observed between Ti-treated and untreated plants (**Figures 8C, E**).

## 4 Discussion

In recent years, an increasing number of reports describe the potential of Ti as a stress-protectant or priming chemical that improves plant responses to various biotic and abiotic stresses (Paret et al., 2013; Karamian et al., 2019; Gohari et al., 2020; Hojjat et al., 2020; Yin et al., 2020; Behnam et al., 2021; Jajor et al., 2021; Mustafa et al., 2021; Satti et al., 2021; Carbajal-Vázquez et al., 2022; Karvar et al., 2022). Ti supply has proven to be efficient in alleviating the stress caused by water deficit and salinity stress, for example. Most of these papers have provided a descriptive framework of the physiological responses of plants treated with Ti, however, the mechanisms by which Ti is transported and sensed by the plant and the molecular responses behind its beneficial effects are still poorly understood. Here, we studied the phenotypic and physiological responses of Arabidopsis to Ti and performed transcriptomic and ionomic profiling at two time-points after Ti exposure.

### 4.1 Ti has positive and negative effects on plant growth depending on Ti concentration

It has been reported that Ti treatment has positive or negative effects on plants depending on the concentration, plant species tested, and in the case of nanoparticles, the type and diameter of the particle (Lyu et al., 2017). We found that Ti has a beneficial effect on plant growth at low concentrations (0.25 and 2.0 mM) and that at higher concentrations (4 to 8mM) Ti inhibits root growth dramatically (**Figure 1A**). The different concentrations that have positive or negative effects on different plant species could be explained by the fact that different species have different Ti uptake rates. Therefore, when working with Ti, the concentration must be optimized based on the research goals and plant species.

It is important to note that the effects of Ti on Arabidopsis fitness and physiology were done under artificial conditions and further tests are required to confirm that observed effects will be similar or the same under field conditions. However, beneficial effects of Ti have been reported under field conditions for plants fertilized with sub-optimal N and P levels. For instance, peppers plants fertilized with 50% N and treated with Ti produced the same biomass as that of plants fertilized with 100% N without Ti treatment (Frutos et al., 1996). Likewise, rice grown in a P-deficient soil produced more biomass when treated with Ti (Zahra et al., 2017). Our results on the effect of Ti treatment on plants fertilized with suboptimal Pi concentrations agree with these reports (**Figure 2**). The root and the shoot of plants treated with 2mM Ti had significantly higher Pi when grown in media with 0.125- and 0.25-mM Pi than untreated controls (**Figure 2**). These results suggest that Ti could indeed be used to make fertilizer use more efficient.

### 4.2 Ti treatment increases the content of some essential nutrients and throttles the uptake of toxic elements

Our results showed that the content of S, P, Cu, Na, Zn, Mn, Fe, and K is higher in the shoot of Ti-treated Arabidopsis plants than in the untreated control (**Figure 3C**). These data are similar to those reported for other plant species showing that Ti enhances the accumulation of different elements in plant tissues (Konishi and Tsuge, 1936; Carvajal and Alcaraz, 1995; Frutos et al., 1996; Wojcik and Wojcik, 2001; Hrubý et al., 2002; Alcaraz-Lopez et al., 2004; Haghighi et al., 2012; Kleiber and Markiewicz, 2013; Servin et al., 2013; Dağhan et al., 2020; Ghazi et al., 2021). The increase of the content of essential elements in Ti-treated Arabidopsis plants correlates well with our RNA-seq data that shows that several genes encoding N, K, S, and divalent cations transporters, as well as P-starvation responsive genes are activated by Ti treatment (**Figure 5A**, **Supplementary Figure S5**). The enhanced Pi content and the reduction in the uptake of toxic elements such as Al, and Cd could be explained by the increased expression of genes encoding organic acid transporters in Ti-treated plants. Organic acid exudation has been shown to enhance Pi uptake and ameliorate Al toxicity by preventing its entry into the root (Hocking, 2001; Barceló and Poschenrieder, 2002; Santos et al., 2014). Our data shows that the root exudates of Ti-treated plants have a higher capacity to solubilize Pi from insoluble compounds and higher chelation of metallic ions (**Supplementary Figure S2**). These results suggest that treatment with 2 mM Ti enhances plant nutrition which is reflected in better growth. However, as we described before, higher Ti concentrations (8 mM) have a drastic effect on Arabidopsis growth, which could be due to a direct toxic effect of Ti or an indirect effect mediated by an exacerbated accumulation of some elements in the plant such as Na or Fe. According to White and Brown (2010), Na, Cl, B, Fe, and Mn are toxic at high concentrations. Considering that in Ti-treated plants the increased Pi accumulation was directly correlated with the amount of Ti applied (**Figure 1C, D**), other elements may be hyperaccumulated at high Ti concentrations. This elemental hyperaccumulation could be responsible, at least in part, of the toxic effect of Ti at high concentrations.

### 4.3 Ti induces transcriptional responses related to ROS and ABA that help Arabidopsis to cope with salt and drought stresses

Gene expression profiling revealed that Ti elicits in Arabidopsis a transcriptional response at multiple levels by upregulating genes involved with defense and tolerance responses to abiotic and biotic stresses that eventually might lead plants to thrive under such stress conditions. Under stress, plants must cope with the generation of reactive oxygen species (ROS) that may lead to DNA, protein, lipid, pigment, carbohydrate, and cellular oxidation/damage (Møller et al., 2007; Miller et al., 2010; Golldack et al., 2014). It has been shown that plants subjected to NaCl stress and treated with Ti, have reduced levels of ROS than plants treated with NaCl only, probably because Ti treatment upregulates antioxidant enzymes genes (Abdel Latef et al., 2018; Gohari et al., 2020). Our transcriptomic data showed that Ti-treated plants have increased transcription of genes encoding peroxidases as well as 30 upregulated DEGs corresponding to the GO category response to oxidative stress in roots (Top 54 GO term, p = 0.00635, **Supplementary Table S7**). These findings suggest that Ti treatment might partially promote the tolerance of plants to abiotic stress by activating genes that protect the plant against the deleterious effects of high ROS production as a consequence of different types of stress. This data is in agreement with previous reports that mention that Ti application enhances the activity of ROS copping enzymes (Bacilieri et al., 2017). We also find enriched GO terms in the Ti treatments related to reactive nitrogen species (RNS), specifically nitric oxide (NO) (GO terms, cellular response to nitric oxide GO:0071732 & nitric oxide biosynthetic process GO:0006809, p = >0.05, **Supplementary Table S7**), which is the most abundant RNS in plants, and play a critical role orchestrating, responses related to biotic and abiotic stresses (Sánchez-Vicente et al., 2019).

Plant responses to drought and salt stresses have been extensively studied because these stresses severely compromise crop yield worldwide (Gamalero and Glick, 2022). Responses to these osmotic stresses are known to be controlled primarily by the ABA-dependent pathway. Endogenous ABA levels increase in response to stress; ABA biosynthesis genes (e.g., AAO3, NCED3, ABA3) are upregulated by a series of transcription factors including NAP, MYB96, WRKY57 (Fujita et al., 2011; Jiang et al., 2012; Yang et al., 2014). Once ABA is synthesized, it is sensed by the ABA sensor, which is composed of PYR/PYL and PP2C proteins (Miyakawa et al., 2013; Yoshida et al., 2014). Sucrose Non-fermenting 1-related protein kinases 2 (SnRK2s) are necessary for ABA signaling and the activation of genes needed to cope with high salinity and drought (Yoshida et al., 2002). These kinases get dephosphorylated by type 2C protein phosphatases (PP2Cs) and remain inactive until osmotic stress (salt or drought) or ABA is perceived by the plant (Golldack et al., 2014; Fernando and Schroeder, 2016). Once ABA or osmotic stress is sensed, PP2Cs are inhibited by the ABA sensor, PYR/PYLs, and ABA, and SnRK2s accumulate in a phosphorylated state (active form). This leads to the activation of genes harboring ABA-responsive elements (ABREs) in their promoters (Fujita et al., 2011; Collin et al., 2021). We found that ABA receptors, PYL4 and PYL6, and SnRK2s, SNF1, SnRK2.10, and SNRK2.4 kinases are upregulated in response to Ti treatment in Arabidopsis. We found that indeed Ti treatment enhances the tolerance of Arabidopsis plants to drought and high salinity treatments (**Figures 6** and **7**). Altogether, these findings suggest that Ti activates the expression of ROS scavenging and ABA-related stress responses genes which eventually lead the plant to cope with salt and drought stresses. Since Ti-treated plants have normal or even enhanced growth compared with control plants, Ti may preactivate these pathways without leading to an unnecessary reduction in the levels of ROS required for cell signaling or the accumulation of deleterious levels of ABA or other hormones that could impair plant growth. We also observed that the pretreatment of plants with Ti before exposure to stress was sufficient to confer higher tolerance to salinity or drought stress. Since the increase of transcripts levels in Ti-treated plants is relatively modest compared to untreated plants and pretreatment with Ti confers tolerance to stress even in the absence of Ti during stress, suggests that Ti could act as a priming molecule that pre-activates the expression of key genes involved in biotic and abiotic stress responses. However, it is also possible that once Ti enters the plant it can cause a biological effect because it remains present in plant tissue for a long time.

RT-qPCR data (**Figures 7C–H**) showed that Ti-treated plants have an enhanced expression of AAO3 and ABI5 when exposed to NaCl, which are critical genes in the ABA biosynthesis and ABA signaling respectively (Barrero et al., 2006; Fujita et al., 2011). This enhanced expression is clear one day after transfer to media containing NaCl and is apparently lost after 7 days of the transfer. These results indicate that a higher level of expression of genes involved in the responses to osmotic stress is critical during the early stages of the response to osmotic stress. The finding that expression of PYL4, ABI5, and AAO3 is significantly higher in seedlings grown in media containing NaCl and Ti than in those grown in media containing NaCl without Ti, confirms that Ti activates the expression of genes involved in osmotic stress, but also that to maintain a higher level of expression requires the continuous presence of Ti.

### 4.4 Ti induces defense responses that protect Arabidopsis against *B. cinerea* infection

Once a pathogen has penetrated the plant, it uses multiple effectors to damage plant tissues and harvest plant nutrients. These effectors, as well as parts of the pathogen itself, and/or parts of the damaged plant cells, are sensed by Pathogen-Associated Molecular Patterns (PAMPs) or Damage-Associated Molecular Patterns (DAMPs) receptors. Examples of these receptors are those belonging to the receptor-like kinases (RLKs) or receptor-like proteins (RLPs) and NBS-LRR families (DeYoung and Innes, 2006; Wang et al., 2020). In our transcriptomic analysis, we discovered that the transcript level of 23 NBS-LRR receptor genes was elevated in Ti-treated plants as compared to control plants (**Figure 8A**). Downstream in the signaling transduction pathway of the LRR receptors, MAPKs play a key role in activating defense responses. Our analyses show that Ti treatment induces an increase in the transcript level of the MAP KINASE KINASE 9 (MKK9), which is important for the synthesis of camalexin and other phytoalexins and secondary metabolites involved with defense response. Interestingly, we also found that PAD4 (PHYTOALLEXIN DEFICIENT 4) is Ti-responsive in roots (**Figure 8B** and **Supplementary Table S3**). PAD4 encodes a lipase-like gene important for SA signaling by modulating phytoalexin synthesis (Glazebrook et al., 1996; Cui et al., 2017). Phytoalexins have been reported to play a crucial role against fungal pathogens (Ahuja et al., 2012; Jeandet et al., 2013). These findings suggest that Ti may activate multiple mechanisms that facilitate the activation of defense responses against pathogens. The upregulation of WES1 in shoots of Ti-treated plants strongly supports the notion that Ti promotes resistance against pathogen, as WES1 overexpression leads to increased camalexin biosynthesis and pathogen resistance (**Figure 8B**). WES1 overexpression also led to increased levels of IAA which could explain the GO categories related to auxins (Zhang et al., 2007; Wang et al., 2012). Noteworthy is the fact that while Ti enhances the resistance against a necrotrophic agent like *B. cinerea*, it could also protect against other species of necrotrophic fungi and even against other pathogenic microbes like biotrophic pathogens as the enhanced expression of NBS-LLR receptors in Ti-treated plants suggest (**Figure 8A**; Paret et al., 2013; Jajor et al., 2021; Satti et al., 2021). It is worth mentioning that there are reports showing that the overexpression of some NBS-LRR genes enhances the plant’s resistance to salt and drought stresses, like the *A. thaliana At*ADR1 or the *Vitis amurensis Va*RGA1 NBS-LRR receptors (Chini et al., 2004; Li et al., 2017). These findings suggest that Ti might activate genes that enhance the tolerance to both biotic and abiotic stress.

### 4.5 Ti and Si beneficial effects on plants may have similar mechanisms of action

It has been reported that Si enhances nutrient uptake and biotic and stress responses in plants in a similar manner as those reported for Ti. Both Si and Ti are tetravalent atoms that cause similar beneficial effects in plants; therefore, it is possible to speculate that both elements enter the plant using similar or common transport systems and signaling pathways. LSI1 transporters, which are members of the aquaporin gene family, have been demonstrated to transport Si in several plant species (Ma and Yamaji, 2015; Vatansever et al., 2017). The finding that the transcripts levels of several NIP aquaporins were increased by Ti-treatment results interesting because they are putative Si transporters (**Figure 5B**) and NIPs are responsive to Ti whereas LSIs to Si (Bokor et al., 2015; Cen et al., 2022). However, the Si transporters in Arabidopsis are still unknown. Therefore, a direct comparison cannot be made at this point. Both Ti and Si alter root exudate profiles (**Figure S2**) (Ghoto et al., 2020) and reduce the uptake of Al and Cd (**Figure 3**) (Mihaličová Malčovská et al., 2014; Pontigo et al., 2017). Moreover, these two elements lead to enhanced drought high salinity tolerance (**Figures 6** and **7**) (Rizwan et al., 2015), and biotic stress (**Figure 8**) (Fauteux et al., 2005). Si also activates genes related to osmotic stresses and biotic stresses related to SA and JA signaling (Mir et al., 2022). These transcriptomic responses are similar to what we found for Arabidopsis treated with Ti.

It was previously proposed that Ti may act through hormesis (Hrubý et al., 2002). Our results show that low Ti concentrations have a positive effect on plant nutrition and tolerance to biotic and abiotic stress, but that at high concentrations Ti becomes deleterious to plant growth. These observations support the hypothesis that Ti acts *via* hormesis. Interestingly, it was recently shown that Si influences plant responses in a hormetic manner (Trejo-Téllez et al., 2020). The parallels between the effects of Si and Ti treatment of plant fitness suggest that these two elements act by common or similar signaling pathways. Further research is needed to determine whether this hypothesis may or may not be rejected.

## 5 Conclusion

Our results confirm that Ti treatment at relatively low doses has a positive effect on growth and the accumulation of essential elements in Arabidopsis seedlings and that a high concentration of Ti has deleterious effects on root and shoot growth. We also report that Ti treatment has a significant effect on the tolerance of Arabidopsis to drought, high salinity, and infection by necrotrophic fungi. Based on the RNA-seq data we propose that Ti promotes plant health and resilience to biotic and abiotic stress by sensitizing the plant and by activating the expression of genes involved in ROS detoxification/signaling, ABA, and SA signaling pathways. This pre-activation, provably a hormetic effect, is led to a level that allows the plant to have a more rapid and protective response to stress but without reaching levels that could be detrimental to the physiology of the plant. Finally, our data suggest that Ti might act as a molecular priming agent that induces internal changes that prepares the plant to respond to diverse environmental stressed. Further research is needed to fully elucidate the mechanisms by which Ti is transported into the plants, the potential sensors that perceive Ti in the plant, and the signaling pathways that activate the myriad of responses activated by Ti in plants.

## Data availability statement

The datasets presented in this study can be found in online repositories. The names of the repository/repositories and accession number(s) can be found below: https://www.ncbi.nlm.nih.gov/, GSE208223.

## Author contributions

FGP-Z conceived the idea, organized, planned, and execute all experiments, analyzed data, wrote and edited the manuscript, and design the figures. KA-H, help with the design and execution of the *Botrytis cinerea* experiments. PM-I and AO-A, help with the execution of drought and salinity experiments, AO-A also help with the implementation of the RNA-seq experiment and RT-qPCR experiments. PM-I also help with the execution of the root exudates experiments. DL-A helped designed figures, analyzed data, and edited the paper together with FGP-Z. LH-E conceived the idea, organized, and planned the experiments, analyzed data, wrote and edited the manuscript, and design the figures. All authors contributed to the article and approved the submitted version.

## Funding

This work was supported in part by grants from the Basic Science program from CONACyT (Grant 00126261), the Governor University Research Initiative program (05-2018) from the State of Texas, and by a Senior Scholar grant from Howard Hughes Medical Institute (Grant 55005946) to LH-E.

## Acknowledgments

FGP-Z is indebted to Consejo Nacional de Ciencia y Tecnología (CONACyT) for the PhD fellowship that allowed the realization of this work.

## Conflict of interest

The authors declare that the research was conducted in the absence of any commercial or financial relationships that could be construed as a potential conflict of interest.

## Publisher’s note

All claims expressed in this article are solely those of the authors and do not necessarily represent those of their affiliated organizations, or those of the publisher, the editors and the reviewers. Any product that may be evaluated in this article, or claim that may be made by its manufacturer, is not guaranteed or endorsed by the publisher.
