# Supplementary material for: Titanium nanoparticles activate a transcriptional response in Arabidopsis that enhances tolerance to low phosphate, osmotic stress and pathogen infection
Source: Front Plant Sci. 2022 Nov 1;13:994523. doi: 10.3389/fpls.2022.994523 (PMC9664069; doi:10.3389/fpls.2022.994523)
Supplement: Supplementary file 2 [file Presentation_1.pptx]

## Slide 1
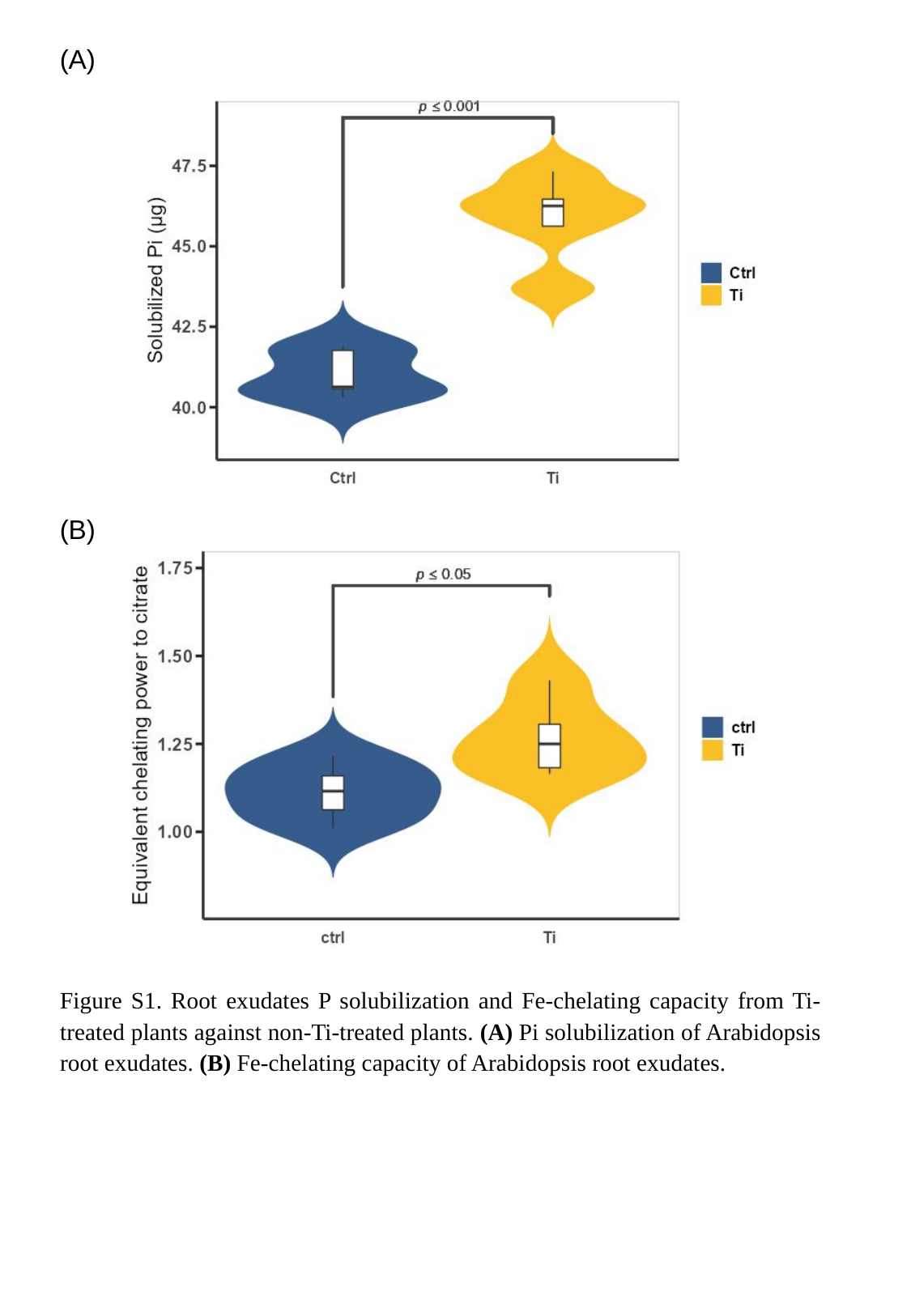

(A)
(B)
Figure S1. Root exudates P solubilization and Fe-chelating capacity from Ti-treated plants against non-Ti-treated plants. (A) Pi solubilization of Arabidopsis root exudates. (B) Fe-chelating capacity of Arabidopsis root exudates.

## Slide 2
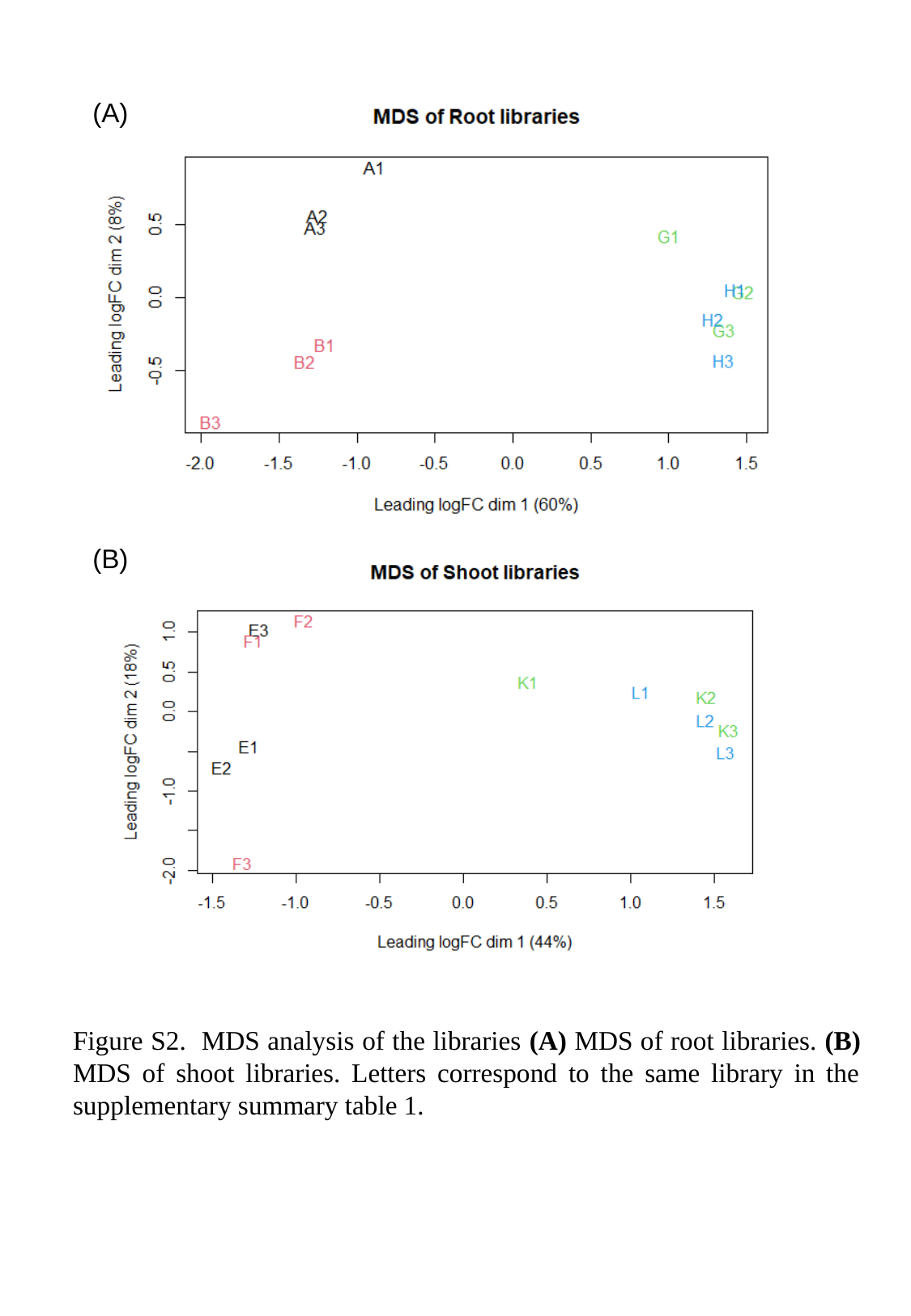

(A)
(B)
Figure S2. MDS analysis of the libraries (A) MDS of root libraries. (B) MDS of shoot libraries. Letters correspond to the same library in the supplementary summary table 1.

## Slide 3
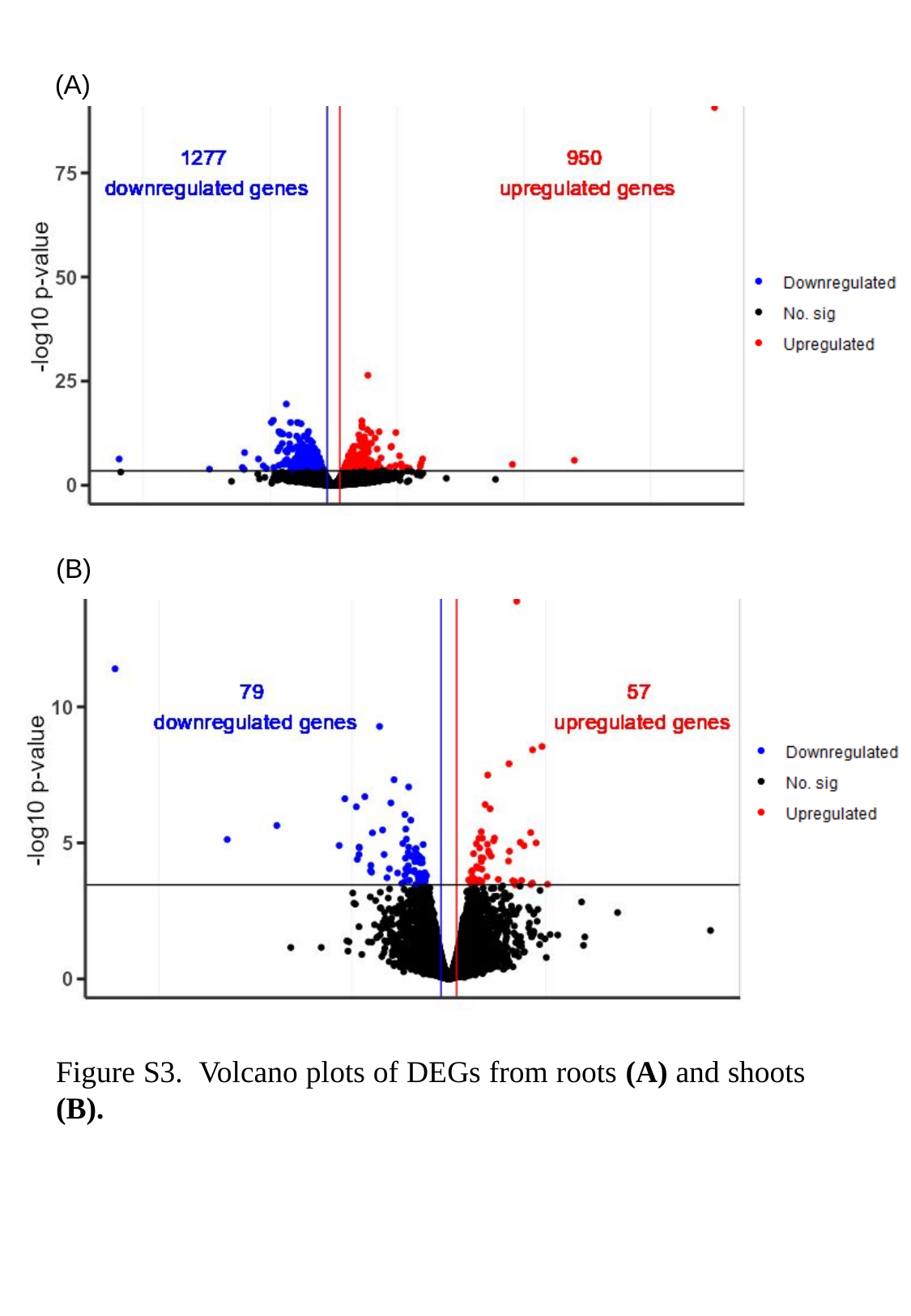

(A)
(B)
Figure S3. Volcano plots of DEGs from roots (A) and shoots (B).

## Slide 4
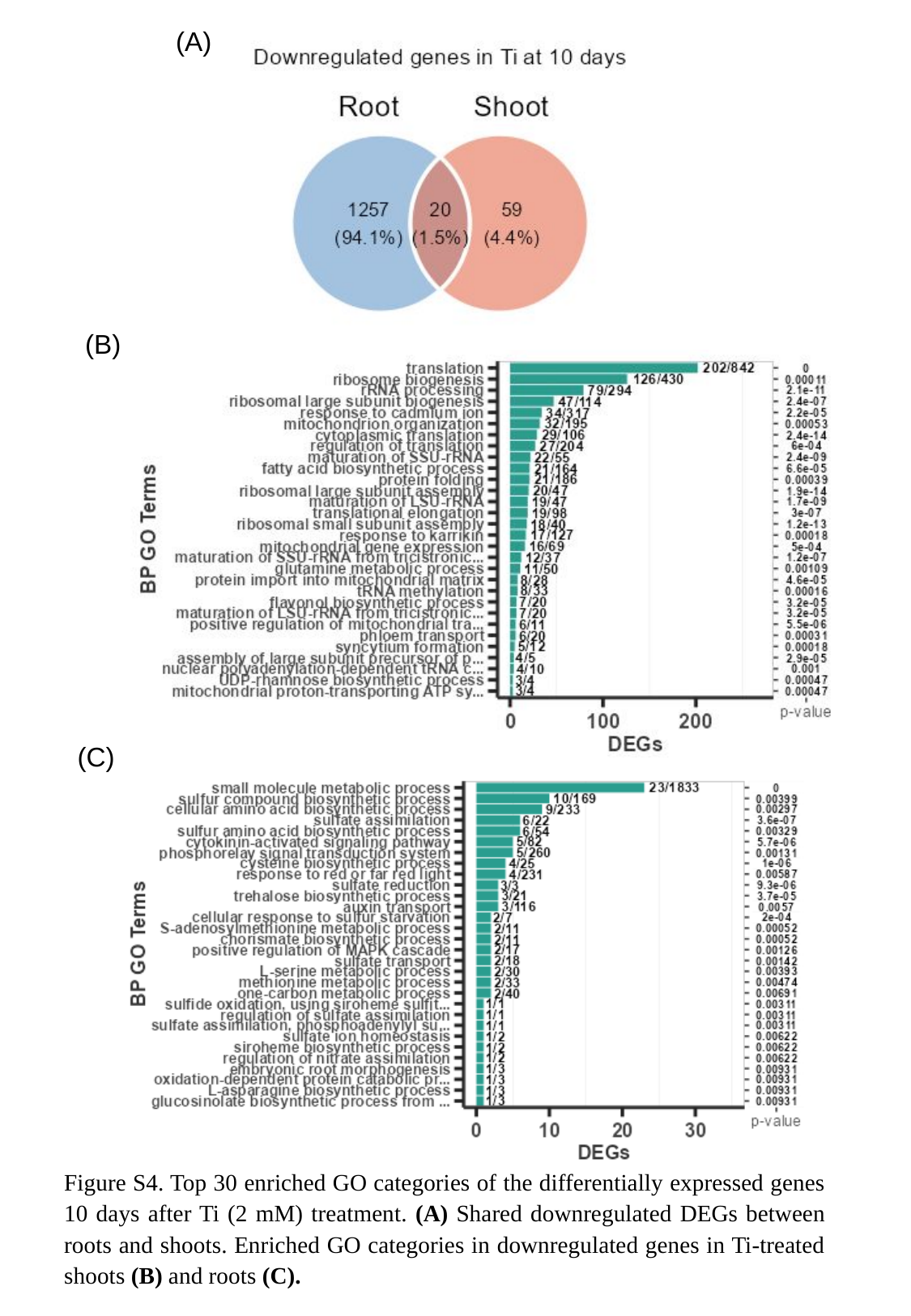

(A)
(B)
(C)
Figure S4. Top 30 enriched GO categories of the differentially expressed genes 10 days after Ti (2 mM) treatment. (A) Shared downregulated DEGs between roots and shoots. Enriched GO categories in downregulated genes in Ti-treated shoots (B) and roots (C).

## Slide 5
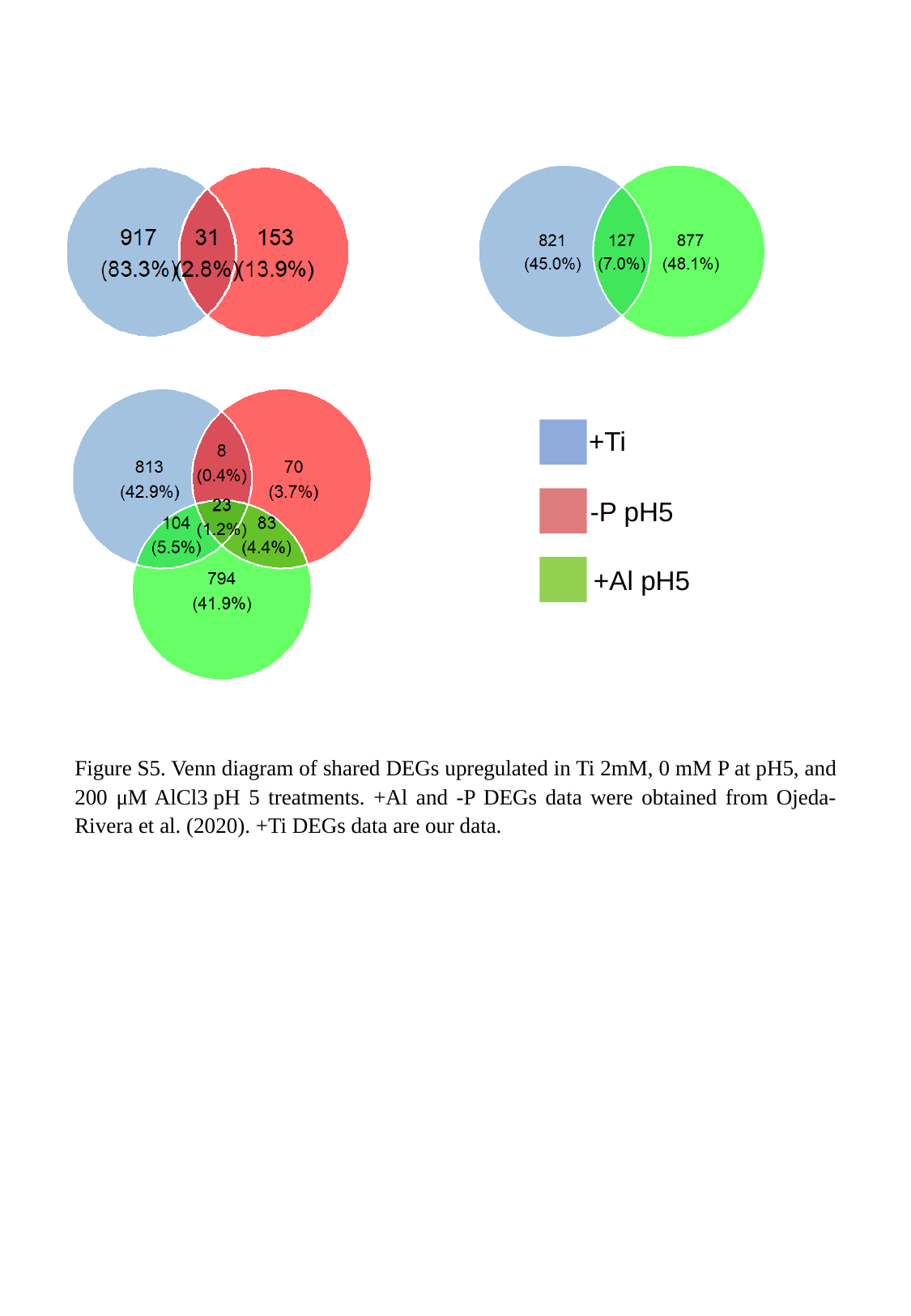

+Ti
-P pH5
+Al pH5
Figure S5. Venn diagram of shared DEGs upregulated in Ti 2mM, 0 mM P at pH5, and 200 μM AlCl3 pH 5 treatments. +Al and -P DEGs data were obtained from Ojeda-Rivera et al. (2020). +Ti DEGs data are our data.
